# On-target IgG hexamerisation driven by a C-terminal IgM tail-piece fusion variant confers augmented complement activation

**DOI:** 10.1038/s42003-021-02513-3

**Published:** 2021-09-02

**Authors:** Joshua M. Sopp, Shirley J. Peters, Tania F. Rowley, Robert J. Oldham, Sonya James, Ian Mockridge, Ruth R. French, Alison Turner, Stephen A. Beers, David P. Humphreys, Mark S. Cragg

**Affiliations:** 1 Antibody and Vaccine Group, Centre for Cancer Immunology, Cancer Sciences, Faculty of Medicine, University of Southampton, Southampton, UK; 2 UCB Pharma, Slough, UK

**Keywords:** Antibody therapy, Complement cascade

## Abstract

The majority of depleting monoclonal antibody (mAb) drugs elicit responses via Fc-FcγR and Fc-C1q interactions. Optimal C1q interaction is achieved through hexameric Fc:Fc interactions at the target cell surface. Herein is described an approach to exploit the tailpiece of the naturally multimeric IgM to augment hexamerisation of IgG. Fusion of the C-terminal tailpiece of IgM promoted spontaneous hIgG hexamer formation, resulting in enhanced C1q recruitment and complement-dependent cytotoxicity (CDC) but with off-target complement activation and reduced in-vivo efficacy. Mutation of the penultimate tailpiece cysteine to serine (C575S) ablated spontaneous hexamer formation, but facilitated reversible hexamer formation after concentration in solution. C575S mutant tailpiece antibodies displayed increased complement activity only after target binding, in-line with the concept of ‘on-target hexamerisation’, whilst retaining efficient in-vivo efficacy and augmented target cell killing in the lymph node. Hence, C575S-tailpiece technology represents an alternative format for promoting on-target hexamerisation and enhanced CDC.

## Abstract (web summary)

Sopp et al describe an approach, which exploits the tailpiece of the naturally multimeric IgM to augment hexamerisation of IgG. Their approach provides a newly engineered format of antibodies for promoting hexamerisation and enhanced complement-dependent cytotoxicity only when cell surface bound.

## Introduction

Monoclonal antibodies (mAb) display utility in the treatment of several cancer indications. The first mAb approved for the treatment of haematologic cancer was the anti-CD20 chimeric human (h)IgG1 rituximab (Rituxan, Mabthera). Rituximab and next-generation anti-CD20 antibodies, such as obinutuzumab, are used front-line in the treatment of CD20+ B-cell lymphomas and leukaemias^1^, employing multiple effector mechanisms to eliminate cancer cells. They can induce cell death directly through Fab-mediated antigen binding (direct cell death (DCD))^2,3^ or Fc-mediated effector functions. The Fc-domain of hIgG1 interacts with Fc gamma receptors (FcγR), where engagement and signalling on immune effector cells elicit antibody-dependent cellular cytotoxicity (ADCC)^4,5^ and/or antibody-dependent cellular phagocytosis (ADCP)^6^. Conversely, mAb can induce cytotoxicity through the recruitment of C1q and subsequent activation of the classical complement cascade. This proteolytic cascade can ultimately result in the insertion of the membrane attack complex and cellular lysis, evoking complement-dependent cytotoxicity (CDC)^7,8^.

Although the clinical approval of new antibodies is continually increasing, many patients remain unresponsive or become resistant to treatment; therefore, developing new therapies that are more efficacious or overcome these resistance mechanisms is key. Emerging antibody therapies are attempting to overcome these problems by expanding the number of immunologically relevant targets and developing alternative means of tumor destruction, such as through checkpoint blockade^9^ and immune stimulation^10^. Nevertheless, these modalities rarely treat >25% of patients successfully^11^, with primary and secondary resistance common and immune toxicities frequently treatment limiting^12^. An alternative approach to overt immune modulation is to augment existing tumor-targeting therapeutics, such as through the use of Fc multimerization technologies.

In this respect, hexameric hIgG1 reagents have been shown to increase mAb efficacy, predominantly as potent CDC-inducing agents^13–16^. The concept of antibody hexamers began with Smith et al.^13^ fusing either the C-terminal tailpiece (tp) peptides of IgA or IgM onto the C-terminus of IgG^14^. These C-terminal tp peptides consist of 18 amino acids with conserved cysteines at the penultimate residue^17,18^, which have been proposed to form disulphide bonds between adjacent tp molecules^19,20^. The IgG tp fusion, therefore, results in the spontaneous covalent multimerisation of the hIgG1. These IgG hexamers exhibited high complement activity in vitro^13,14^. A similar enhancement of CDC can be produced using hIgG1 containing hexamerisation enhancing Fc mutations. These mAb are monomeric in solution but cluster at the cell surface after antigen binding to form ordered, but non-covalent hexamers^21^. This increase in complement activity has been attributed to elevated avidity of the IgG hexamer for the six-headed globular protein, C1q^22^ as exemplified by the E430G and E345R mutations^15,16^. Elegant structural data support the association of each head group of C1q with a single Fc molecule of both hexameric IgM^23^ and hexameric IgG^24^. Alternatively, complement activities can be improved by selectively enhancing the affinity for C1q, resulting in highly potent CDC-evoking mAb^25,26^, or through IgG1/IgG3 chimerisation^27^.

CDC-enhanced mAb has the potential to improve future therapeutics. Herein, we created an alternative hexamerisation approach through the fusion of a mutated form of the IgM tp (μtp) at the C-terminus of hIgG Fc. Mutagenesis of the penultimate cysteine of the μtp to a serine ablated spontaneous and stable hexamerisation in favour of hIgG1 monomers in solution, but with an enhanced propensity for non-covalent Fc–Fc interactions, leading to multimerisation. The engineered hIgG μtp C575S offers enhanced complement activity and maintains FcγR-effector mediated mechanisms in vitro, with whole-blood B-cell depletion comparable to wild-type hIgG1. In addition, in vivo efficacy, safety and half-life were at least equivalent to wild-type hIgG1, demonstrating hIgG1 μtp C575S as a potential in vitro and in vivo format for enhanced CDC.

## Results

### Engineering of hexamerisation-enhanced anti-CD20 hIgG1

**Fig. 1:**
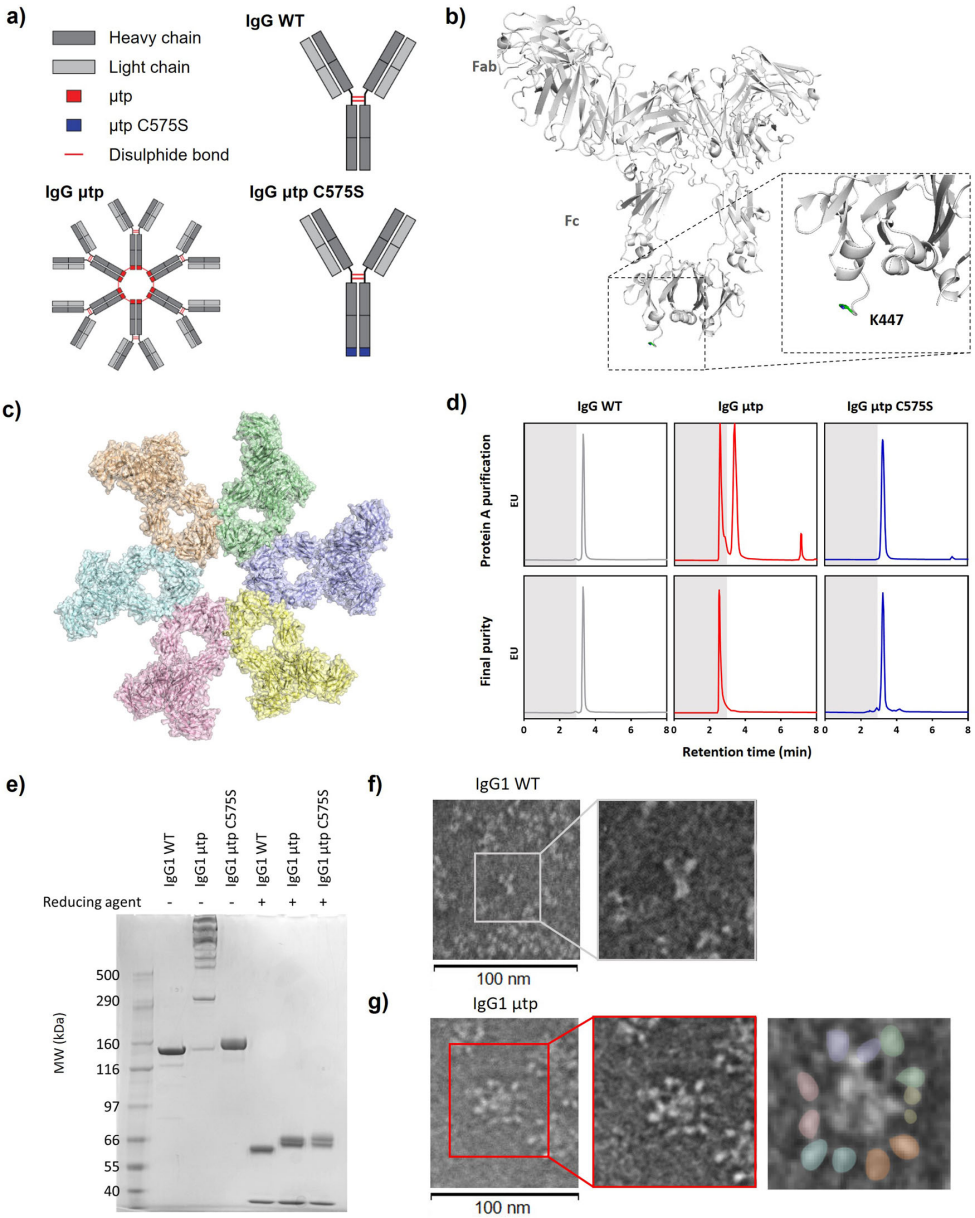
Generation and characterisation of hIgG1 μtp fusion mAb. **a** Schematic of the hIgG μtp hexamer and hIgG1 μtp C575S monomer mAb. **b** Structure of IgG-b12 hexamer (PDB: 1HZH). The C-terminal lysine (K447) is highlighted in the insert and coloured green. **c** Hexameric IgG structure observed in the crystal packing arrangement of IgG-b12 (PDB: 1HZH). **d** CHO produced hIgG1 μtp fusion mAb were purified using protein A chromatography followed by size-exclusion chromatography, representative SE-UPLC chromatograms shown for rituximab hIgG1 wild-type (grey), rituximab hIgG1 µtp pre-formed hexamer (red), and rituximab hIgG1 µtp C575S on-target hexamer (blue). **e** 3–8% Tris-acetate SDS-PAGE analysis to assess the size of the purified hIgG1 μtp fusion mAb in non-reduced and reduced forms. **f**, **g** For negative stain electron microscopy, mAb were applied to glow discharged electron microscopy grids stained with 2% uranyl acetate solution. Images were collected using a Hitachi HT7700 Transmission Electron microscope at ×80,000 magnification, single images were processed using Adobe Photoshop. Images shown depict **f** rituximab hIgG1 wild-type and **g** rituximab hIgG1 µtp pre-formed hexamer. Rituximab hIgG1 µtp pre-formed hexamer with Fab pairs highlighted as separate colours shown separately. Scale bar 100 nm.

In order to generate and evaluate hIgG1 hexamer antibodies, we fused the μtp of IgM to the C-terminus of the hIgG1 heavy chain (hIgG μtp; Fig. 1a and b) in rituximab constructs. hIgG1 is arranged as a hexamer in the crystal packing of IgG1-b12 (Protein Data Bank entry 1HZH)^28^, with interactions observed at the CH2:CH3 interface (Fig. 1c), indicating this tail-to-tail arrangement may be naturally favoured. To examine the importance of the penultimate cysteine residue (denoted herein as C575) of the μtp for stable hexamerisation, we mutated it to serine by site-directed mutagenesis (hIgG μtp C575S; Fig. 1a). Serine was selected because it has similar physiochemical properties to cysteine (polar and isosteric). Hence, serine may be considered the most conservative alternative to cysteine from both production science and perceived in vivo immunogenicity perspectives. These molecules were expressed in CHO-SXE cells, providing an average yield of 398.4 ± 8.8 (±SD) mg/L for the rituximab hIgG1 μtp C575S and 414.3 ± 114.9 (±SD) mg/L for the rituximab hIgG1 μtp pre-formed hexamer, compared with 315.5 ± 81.0 (±SD) mg/L for the native hIgG1, demonstrating that the addition of the μtp C575S or hIgG μtp does not adversely affect protein yield (Table 1). Protein A purification followed by size-exclusion ultra-high performance liquid chromatography (SE-UHPLC) analysis showed that the hIgG1 μtp constructs yielded two species representing monomers and hexamers, as judged by molecular weight controls. Size-exclusion chromatography was therefore used to produce a pure hexamer product (Fig. 1d). Similar analysis of the hIgG1 μtp C575S construct demonstrated a single species and profile identical to native monomeric hIgG1, indicating that the C575 was critical for spontaneous hexamerisation (Fig. 1d). Final purification involved a buffer exchange or size-exclusion step and was identical to native hIgG1. The average yield for the purified hIgG1 μtp C575S molecule was 211.3 ± 95.4 (±SD) mg/L, compared with 245.8 ± 73.5 (±SD) mg/L for rituximab hIgG1 wild-type and 40.8 ± 20.7 (±SD) mg/L for the hIgG1 μtp pre-formed hexamer. The lower recovery of pre-formed hexamer is largely a reflection of the use of SEC to remove non-hexamer species (Table 1). To indicate the broad applicability of this approach, the same three mAb formats were produced in the context of four different V regions (Supplementary Table 1) with the results showing the μtp C575S and μtp peptide additions do not overtly impact protein expression. Nevertheless, the removal of non-hexamers from the μtp constructs consistently resulted in a lower final yield, illustrating one of the challenges of using such molecules clinically. Together, these data demonstrate that mAb production is reproducible and robust across μtp formats and amenable to multiple mAb V regions.

**Table 1:** Estimated expression yield and calculated purified yield for rituximab IgG1 μtp constructs.

mAb construct	Expressed yield* (mg/L)	Purified yield^+^ (mg/L)	% of expressed yield
Rituximab IgG1 wild-type	414.3 ± 114.9	245.8 ± 73.5	59.3
Rituximab IgG1 μtp C575S	398.4 ± 8.8	211.3 ± 95.4	53.0
Rituximab IgG1 μtp	315.5 ± 81.0	40.8 ± 20.7	12.9

*Expression yield (mg/L) was calculated post expression by protein G HPLC. ^+^Purified yield (mg/L) was calculated post size-exclusion chromatography. Data shown are mean ± SD of expression and purifications of the three different mAb formats (*n* = 3 different preparations).

**Fig. 2:**
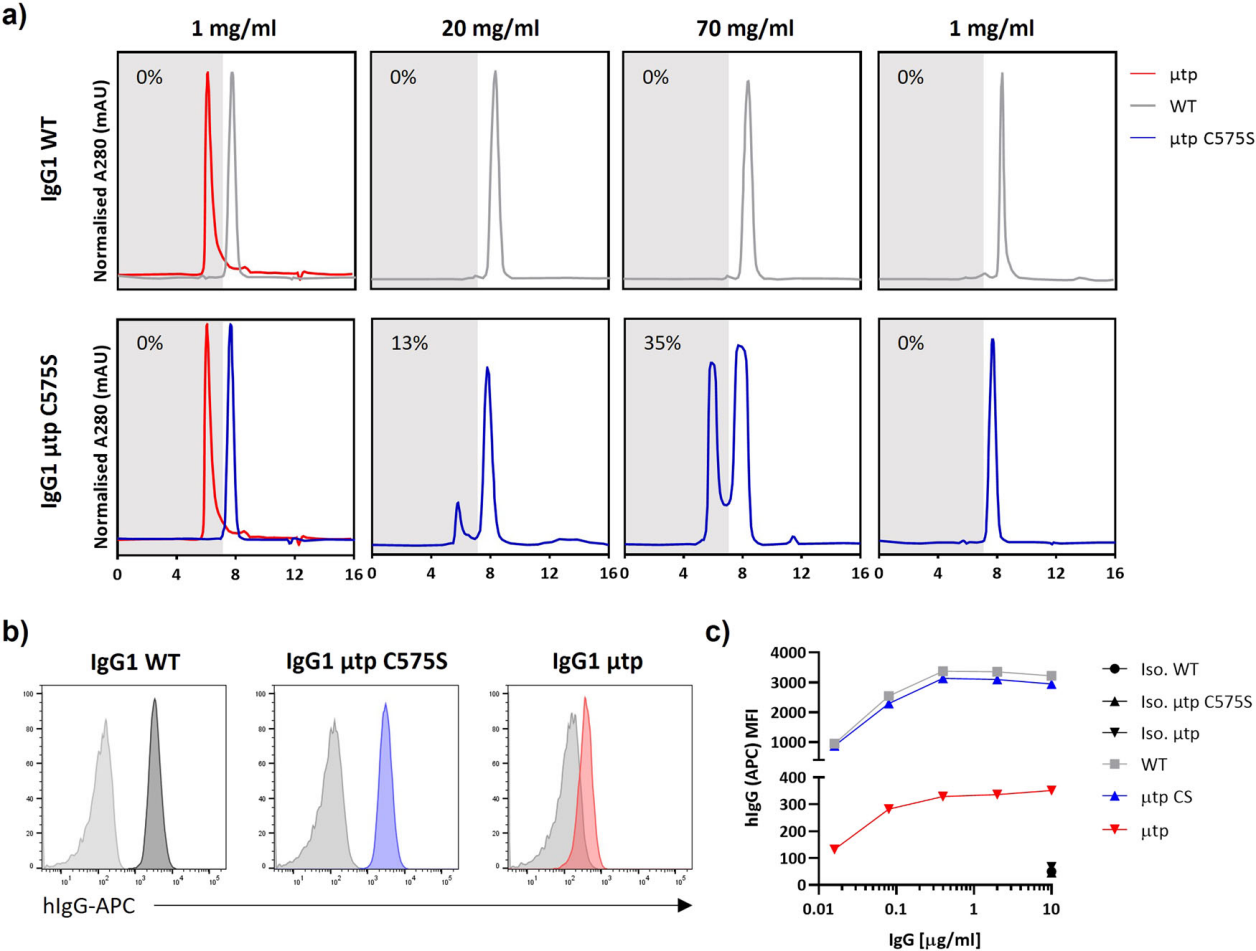
Assessment of hexamerisation enhancement and antigen binding of IgG1 μtp constructs. **a** Rituximab mAb constructs were concentrated up to 70 mg/ml and diluted to the required concentrations and analysed by SE-HPLC for the percentage of monomeric and multimeric species. Shown are SE-HPLC chromatagrams of hIgG1 wild-type and hIgG1 μtp C575S overlaid with purified hIgG1 μtp pre-formed hexamer trace prior to concentration, antibody concentrated to 20 mg/ml and 70 mg/ml, and post dilution back to 1 mg/ml. **b** Ramos cells were opsonised with rituximab hIgG1 μtp mAb at 10 μg/ml and binding measured by secondary anti-human Fc-APC-labelled antibody. Solid grey histograms indicate matched Herceptin hIgG wild-type and hIgG μtp isotype control mAb. **c** Antibody binding (MFI) over a concentration range of rituximab hIgG μtp and isotype control hIgG μtp mAb binding Ramos cells (representative data shown).

Analysis by sodium dodecyl sulphate–polyacrylamide gel electrophoresis (SDS-PAGE) indicated a monomeric species with the hIgG μtp C575S molecule, comparable to hIgG1 wild-type (Fig. 1e). Under non-reducing SDS-PAGE conditions, the hIgG1 μtp exhibited a ladder of various sizes up to a predicted hexamer (Fig. 1e), which was not observed under solution analysis with SEC. The laddering observed is consistent with that seen previously with Fc-hIgG µtp^29^. Both hIgG μtp C575S and hIgG1 μtp molecules were subsequently analysed by size-exclusion chromatography-multi-angle light scattering (SEC-MALS) to determine absolute molecular weights. These were calculated to be 154 kDa and 871 kDa, respectively, compared with 149 kDa for hIgG1 wild-type. The molecular weight of 871 kDa corresponded to six hIgG1 μtp monomers. Further to SEC-MALS, negative stain EM demonstrated that the purified hIgG1 μtp construct was arranged as a hexamer (Fig. 1g). Subsequently, we used a solution-based concentration assay to assess if the hIgG1 μtp C575S molecule exhibited an increased propensity to undergo concentration-dependent hexamerisation, observed as a shift from monomer to higher molecular weight species (multimer) by SEC. At 20 mg/ml the hIgG1 μtp C575S exhibited 13% multimer species which rose to 35% at 70 mg/ml (Fig. 2a). In comparison, the hIgG1 wild-type demonstrated no change in multimerisation at either concentration. The multimeric peak aligned with the retention time for the hIgG1 μtp hexamer. This indicates the μtp C575S has the propensity to self-associate in solution in a concentration-dependent manner. Moreover, this association was reversible, disappearing after dilution to 1 mg/ml (Fig. 2a). Comparable results were observed with trastuzumab hIgG1 μtp C575S, demonstrating this propensity to self-associate at high concentrations was independent of the V-region (Supplementary Figure 1). Subsequently, the anti-CD20 hIgG1 μtp constructs were assessed for target binding. All anti-CD20 constructs were shown to bind CD20+ cells demonstrating that μtp and μtp C575S fusions do not significantly impair F(ab)-mediated binding (Fig. 2b and c).

### Analysis of complement activation

**Fig. 3:**
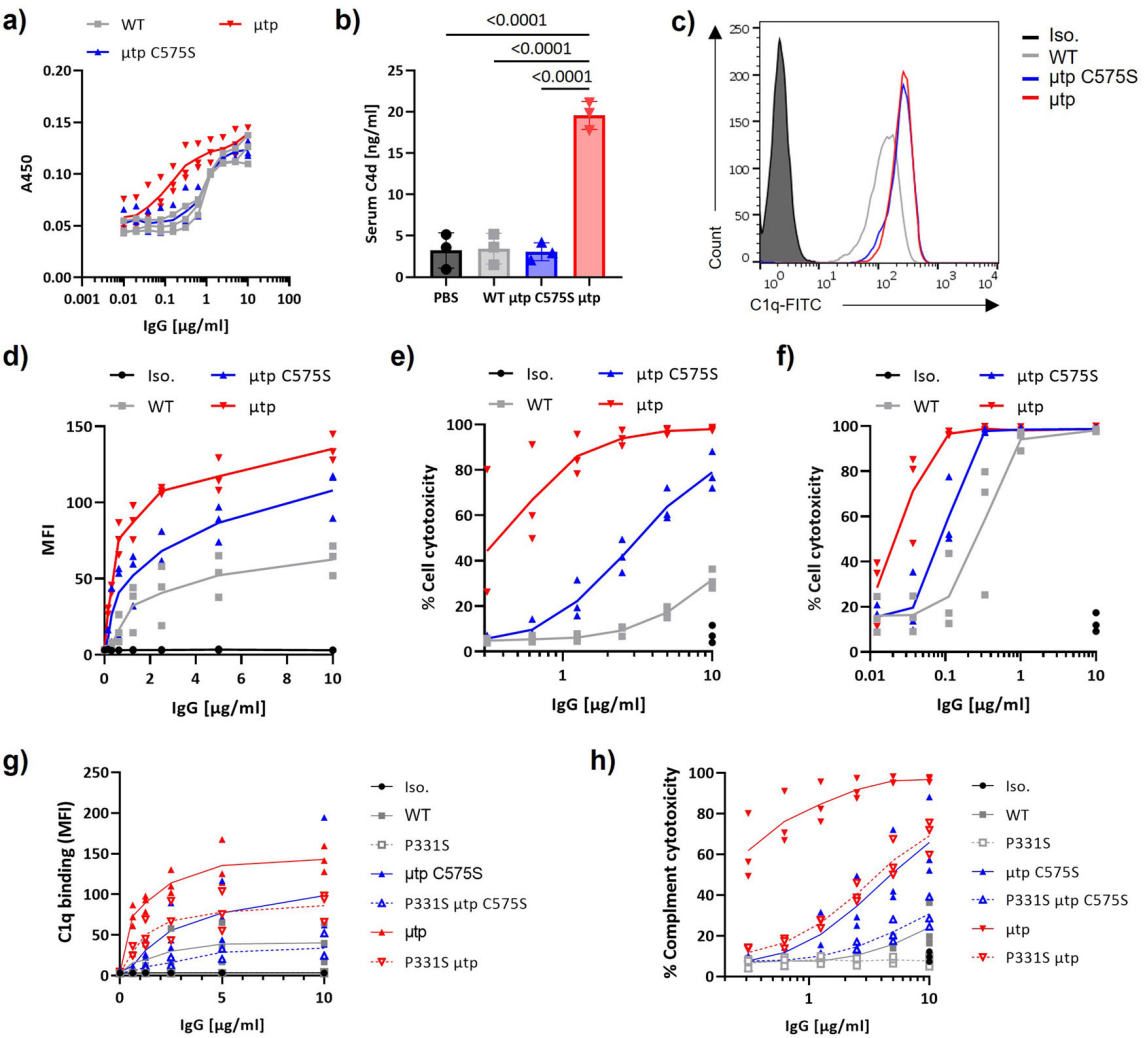
CDC enhancement of hIgG1 μtp C575S and μtp pre-formed hexamer. **a** ELISA plates were coated with rituximab hIgG1 μtp constructs at various concentrations and purified human C1q (2 μg/ml) added. Bound C1q was detected with a goat-anti-C1q, followed by an anti-goat-HRP-conjugated antibody. Data show absorbance at 450 nm (*n* = 3). **b** Fluid-phase C4d was measured in human serum after 1 h incubation with various rituximab hIgG1 constructs at 100 μg/ml. Data show C4d concentration of individual donors (*n* = 3). **c** Ramos cells were opsonised with rituximab hIgG1 constructs at 10 μg/ml, followed by incubation with 2 μg/ml human C1q. Deposition of C1q was analysed using an anti-C1q-FITC antibody. **d** C1q deposition on Ramos cells opsonized with 10–0.15 µg/ml rituximab hIgG1 constructs (*n* = 3). **e**, **f** CD20+ cell lines were opsonised with rituximab hIgG1 μtp constructs at a range of concentrations and incubated with NHS (20 % V/V). Cell death was examined as the percentage of PI-positive cells by flow cytometry. Complement mediated cell death was assessed on Raji (**e**; *n* = 3) or Ramos (**f**; *n* = 3). **g** C1q recruitment to Ramos cells investigated using rituximab hIgG1 µtp P331S constructs (*n* = 3). **h** CDC activity induced by rituximab hIgG1 µtp P331S constructs (*n* = 3). Individual data points and mean  shown from biologically independent experiments. Statistical analysis was carried out by one-way ANOVA.

To determine whether the propensity to hexamerise conferred increased C1q binding, we performed enzyme-linked immunosorbent assay (ELISA) with plate-coated hIgG1 constructs. Only the hIgG1 μtp hexamer exhibited increased C1q binding above wild-type hIgG1 (Fig. 3a, Supplementary Figure 2a), presumably owing to enhanced avidity for C1q. The hIgG1 μtp C575S had comparable binding to hIgG1 wild-type (Fig. 3a). Next, we assessed the capacity to elicit spontaneous complement activation, measuring the production of C4d in fresh human serum (in the absence of target cells) over 1 h at 37°C (Fig. 3b). The addition of the hIgG1 μtp C575S and wild-type hIgG1 did not elicit elevated C4d. However, the hIgG1 μtp hexamer caused a significant increase in C4d compared with hIgG1 wild-type and hIgG1 μtp C575S. These results suggest that only the pre-formed hIgG1 μtp hexamer has increased avidity for C1q, resulting in spontaneous solution-phase initiation of the complement cascade, independent of target binding.

A cell-based C1q recruitment assay was next used to determine C1q-binding differences between the constructs after antigen binding on a target cell. Ramos cells were opsonised with different mAb variants and then incubated with purified human C1q, before detection with anti-C1q-FITC (Fig. 3c). The hIgG1 μtp C575S and hIgG1 μtp both exhibited increased C1q recruitment to the target cell surface compared with wild-type hIgG1, in a dose-dependent manner (Fig. 3d). Subsequently, we evaluated whether these differences translated to preferential CDC in two different cell lines with differing complement sensitivity (Fig. 3e, f, Supplementary Figure 3). Increases in CDC were seen with both the μtp C575S and pre-formed hIgG1 μtp hexamer over the wild-type hIgG1, with the effects most impressive on the more CDC-resistant Raji cell line (Fig. 3e), which expresses physiologically relevant levels of CD55 and CD59^30^. These results demonstrate a direct association between cell surface C1q recruitment and CDC activity, and are in-line with the concept of on-target hexamerisation for the μtp C575S construct.

In addition to demonstrating that the μtp and μtp C575S formats could augment cell surface C1q binding and CDC with rituximab hIgG1, we also investigated whether they could overcome low-affinity C1q interactions. The P331S mutation is known to abrogate C1q binding in hIgG1^31^. Incorporation of the P331S mutation into rituximab hIgG1 μtp constructs reduced the C1q binding of wild-type hIgG1, hIgG1 μtp and hIgG1 μtp C575S in ELISA, most notably for the μtp hexamer (Supplementary Figure 3b–d and 4). It also completely abolished the C1q cell surface binding and CDC activity for hIgG1 wild-type, whereas the μtp C575S P331S and μtp P331S retained some, albeit reduced, C1q recruitment, exhibiting a 53 and 29% drop in CDC killing, respectively (Fig. 3g and h). These data indicate that the μtp and μtp C575S formats can overcome low-affinity C1q interactions by increasing C1q avidity and that this accounts for their enhanced CDC.

**Fig. 4:**
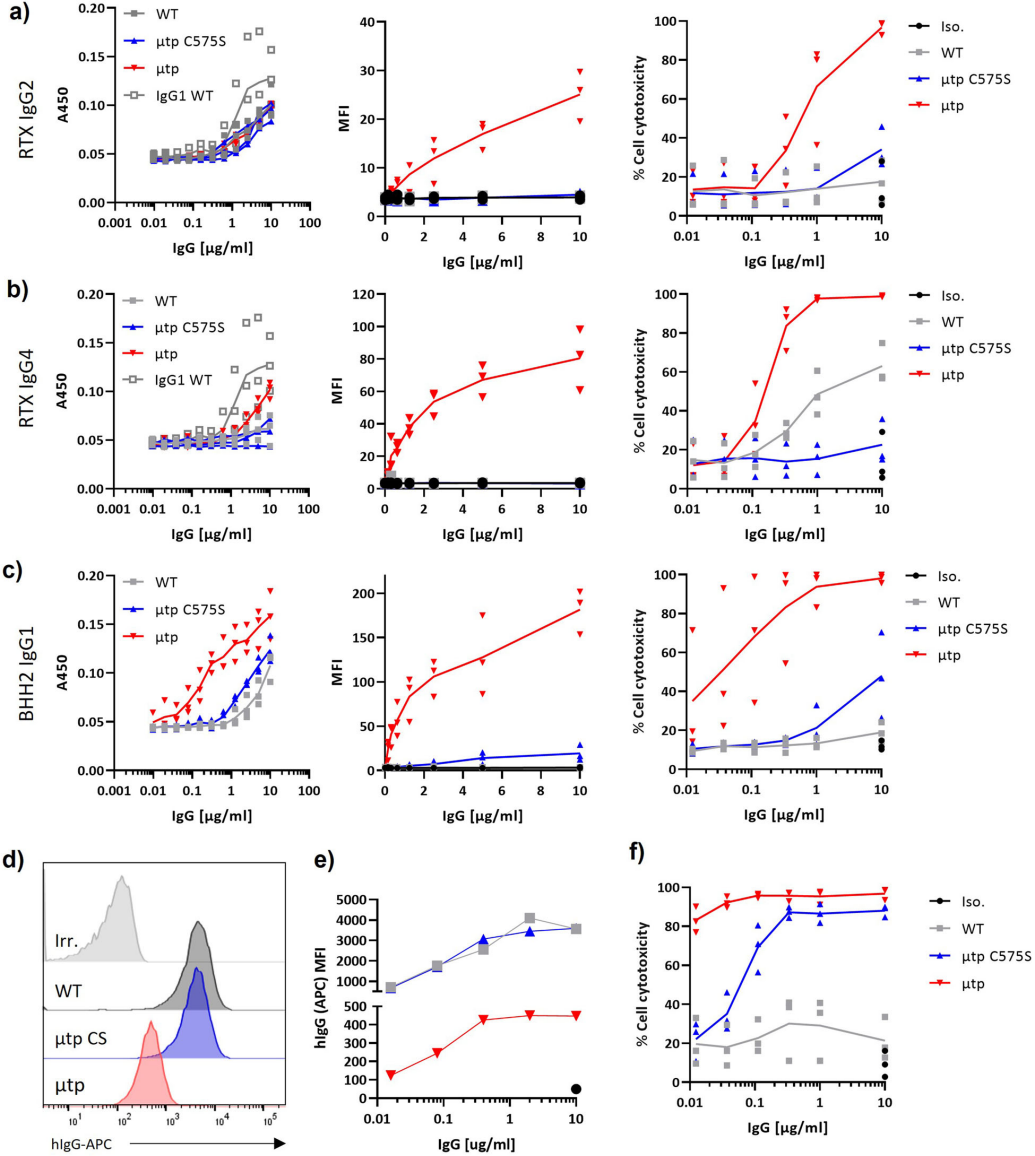
CDC enhancement is also observed with different hIgG isotypes and another CD20 epitope. C1q binding was measured by ELISA. ELISA plates were coated with hIgG µtp constructs at various concentrations and purified human C1q (2 μg/ml) added. Bound C1q was detected with a goat-anti-C1q, followed by an anti-goat-HRP-conjugated antibody. Data show absorbance at 450 nm. C1q cell recruitment was assessed by opsonising Ramos cells with hIgG µtp constructs, followed by incubation with 2 μg/ml human C1q. Deposition of C1q was analysed with an anti-C1q-FITC antibody. CDC-induced cell death was assessed by opsonising Ramos cell with 10–0.15 µg/ml hIgG µtp constructs and incubated with NHS (20 % V/V). Cell death was examined as the percentage of PI-positive cells by flow cytometry. Results are shown for **a** rituximab hIgG2, **b** rituximab hIgG4, and **c** BHH2 hIgG1 µtp constructs. **d** Ramos cells were opsonised with daratumumab (anti-CD38) hIgG1 μtp mAb at 10 μg/ml and binding measured by secondary anti-human Fc-APC-labelled antibody. Solid grey histograms indicate trastuzumab hIgG isotype control mAb. **e** Antibody binding (MFI) over a concentration range (*n* = 1). **f** CDC-induced cell death was assessed for daratumumab μtp antibodies after opsonisation of Ramos cells. Individual data points and mean shown from biologically independent experiments (*n* = 3).

Having established these enhanced properties for hIgG1, we next explored whether other isotypes could be similarly augmented and assessed rituximab hIgG2 and hIgG4 μtp molecules. All molecules were successfully produced, despite the purified yield for all rituximab hIgG2 and IgG4 reagents being low (Supplementary Table 2). These were then assessed for their ability to bind C1q and capture it at the cell surface, secondary to evoking CDC. Both hIgG2 and especially IgG4 are defined by a paucity of binding to C1q^32^. This was confirmed in our C1q ELISA with wild-type hIgG4, showing no appreciable binding and hIgG2 exhibiting lower levels than hIgG1 (Fig. 4a and b, Supplementary Figure 3e and f). Although neither hIgG2 μtp C575S nor μtp demonstrated enhanced C1q binding by ELISA, the hIgG2 μtp molecule recruited higher levels of C1q to the cell surface, and both formats displayed augmented CDC activity against Ramos cells, in particular, the hIgG2 μtp pre-formed hexamer that lysed 100% of targets at 10 µg/ml, compared with no increase above baseline for wild-type hIgG2 (Fig. 4a). The hIgG4 μtp C575S antibody demonstrated negligible binding of C1q, similar to hIgG4 wild-type, but the hIgG4 μtp pre-formed hexamer exhibited enhanced binding (Fig. 4b). This enhanced binding correlated with efficient recruitment of C1q at the cell surface and robust CDC. Interestingly, the hIgG4 μtp C575S displayed a loss of CDC activity compared with the hIgG4 wild-type (Fig. 4b), perhaps associated with a small decrease in C1q binding observed by ELISA.

Rituximab is a so-called type I anti-CD20 mAb and as such, known to redistribute and cluster CD20 within the plasma membrane to evoke efficient CDC^30,33^. Therefore, to assess the broader applicability of our findings, we generated a second series of anti-CD20 μtp mAbs based upon the type II mAb BHH2. BHH2 is related to the glycomodified mAb obinutuzumab, which evokes low levels of CDC^34^ and which we have previously shown to exhibit classical type II behaviour, lacking clustering of CD20 and internalisation^35^. BHH2 hIgG1 μtp C575S demonstrated a slight increase in C1q binding over the hIgG1 wild-type, but the μtp pre-formed hexamer had much greater binding as observed for rituximab (Fig. 4c, Supplementary Figure 2g). The μtp C575S also evoked a modest increase in C1q recruitment and CDC activity when targeting Ramos cells, whereas the μtp pre-formed hexamer demonstrated an increase in both C1q recruitment and CDC activity (Fig. 4c), comparable to that seen with rituximab.

These results clearly demonstrated that the hIgG µtp and µtp C575S technology could augment anti-CD20 mAb-mediated CDC against haematological cell targets. To assess the capacity of the hIgG µtp and µtp C575S technology to augment CDC against other targets, the V regions of the anti-CD38 mAb daratumumab were incorporated into the hIgG1 μtp and μtp C575S backbones. These mAb were efficiently produced as before with retained cell surface binding, albeit with lower detection of the μtp molecule, potentially owing to steric hindrance of the detecting anti-hIgG-APC (Fig. 4d and e). The functionality of this binding was demonstrated in the following CDC assay, which showed a highly effective lysis of the target Ramos cells with the μtp hexamer compared with the hIgG1 wild-type antibody. Lower, but still highly effective lysis was also shown with the μtp C575S format (Fig. 4f). These results clearly demonstrated that the hIgG µtp and µtp C575S technology could augment mAb-mediated CDC against multiple haematological cell targets. To assess the utility of this approach for solid tumour targets, the μtp technology was applied to the anti-HER-2 antibody trastuzumab using SK-BR-3 cells as a solid tumor target. SK-BR-3 were resistant to wild-type hIgG1 trastuzumab. Nevertheless, the μtp pre-formed hexamer format was able to overcome this, producing appreciable levels of CDC at the top concentrations (Supplementary Figure 5).

### Analysis of FcγR-mediated interactions and effector functions

Having established the ability of the μtp technology to augment complement activity, we assessed interactions with FcγR, first exploring whether FcγR-binding affinity and/or avidity was affected using surface plasmon resonance (SPR). The various mAb constructs were immobilised to a Biacore sensor chip and recombinant hFcγR was passed over at various concentrations. Binding affinities to hFcγR were largely unaffected by the addition of the μtp C575S or μtp (Supplementary Figure 6). The binding of the μtp constructs to FcγR was also assessed using CHO cells stably expressing human FcγR^36^ (Supplementary Figure 7). Binding to FcγRI was similar for all antibody formats. In contrast to the SPR analysis, the hIgG1 μtp pre-formed hexamer displayed a higher level of binding to all low-affinity FcγR when compared with the monomeric hIgG1 wild-type and μtp C575S, most likely owing to higher avidity interaction with FcγR. However, the hIgG1 μtp pre-formed hexamer did demonstrate some evidence of non-specific binding to CHO cells not expressing FcγR, and not all CHO cell lines expressed high levels of FcγR.

**Fig. 5:**
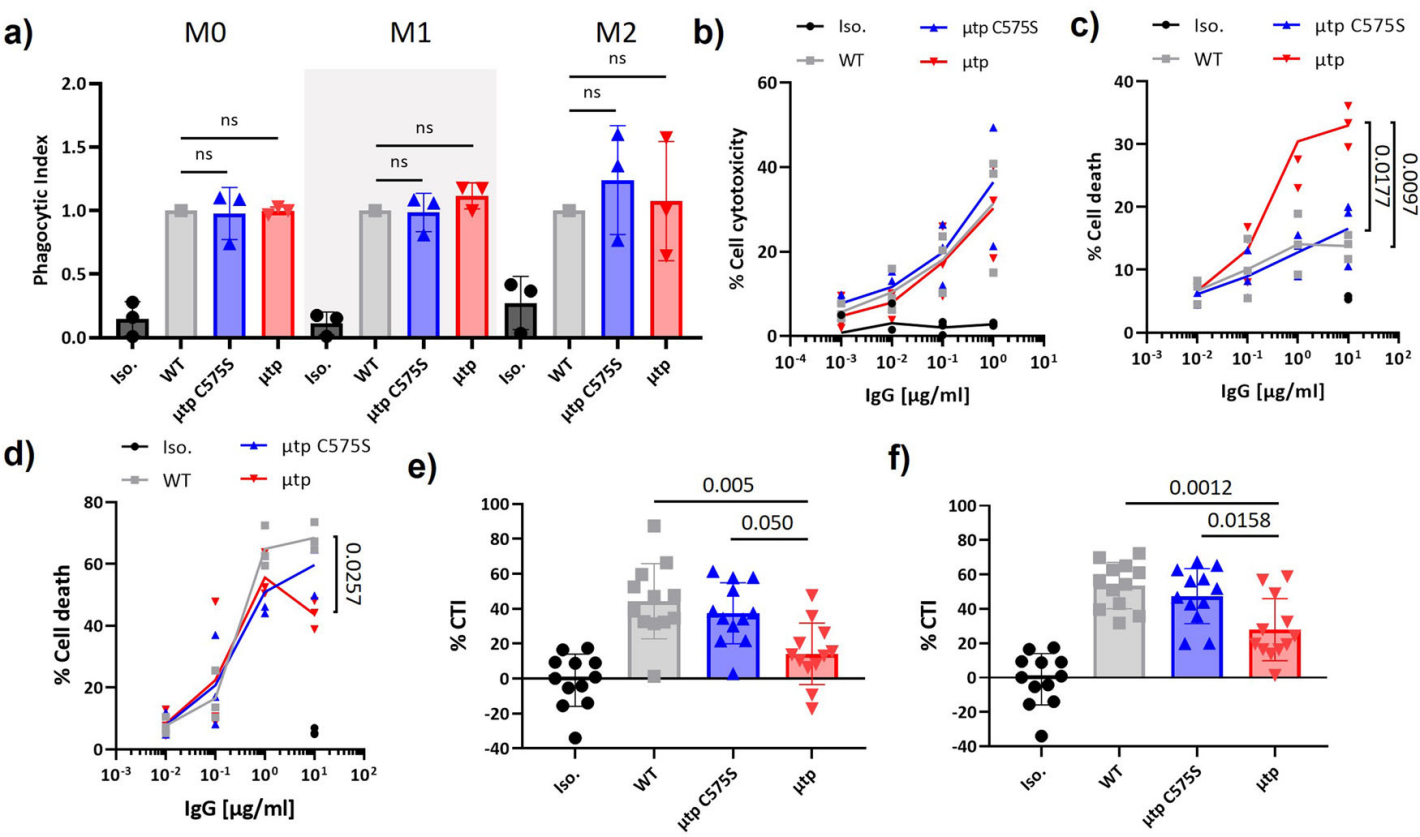
FcγR-mediated effector functions in vitro and B-cell depletion in human whole blood of rituximab IgG1 μtp fusion mAb. **a** CFSE-labelled CLL PBMCs were opsonised with 0.5 μg/ml IgG1 μtp constructs and co-cultured with human MDMs. Phagocytosis was measured by flow cytometry assessing double-positive CFSE and FcγRIII macrophages. Phagocytosis was examined in macrophages skewed in vitro to M0, M1 (Pam3SK4 stimulation), and M2 (IL4/IL13 stimulation) polarisation states. Data show the phagocytic index mean and SD from independent experiments (*n* = 3). Statistical analysis was calculated using one-way ANOVA. **b** Calcein labelled Ramos cells were opsonised with rituximab hIgG1 μtp constructs and incubated with freshly purified PBMCs. The calcein release from cells was used to calculate the % of cell cytotoxicity. Individual data points and mean plotted from independent experiments (*n* = 3). **c**, **d** Raji target cells were incubated with either **c** rituximab hIgG1 μtp fusion mAb or **d** BHH2 hIgG1 μtp fusion mAb for 24 h at 37°C. DCD was assessed for double-positive annexin-V and PI by flow cytometry. Results show the individual data points and mean from independent experiments (*n* = 3). Statistics were calculated using two-way ANOVA with repeated measures. **e**, **f** Fresh peripheral human blood was incubated with IgG1 μtp fusion mAb (1 µg/ml) for 24 h at 37°C. B-cell depletion (cytotoxicity index [CTI]) was calculated by the ratio of B cells to T cells using flow cytometry. Results show CTI for **e** rituximab IgG1 μtp fusion mAb and **f** BHH2 IgG1 μtp fusion mAb. Data are plotted as mean and SD, individual points represent biologically independent donors (*n* = 12). Statistical analysis was carried out by one-way ANOVA.

We next assessed their ability to initiate FcγR-mediated effector functions. ADCP assays with human monocyte-derived macrophages (MDM) and human CLL target cells were used to determine the phagocytic potential of rituximab hIgG1 μtp fusion mAb, by observing double-positive CFSE target cells and FcγRIII + macrophages (Supplementary Figure 8a). Both hIgG1 μtp pre-formed hexamer and hIgG1 μtp C575S constructs retained activity equivalent to wild-type hIgG1, irrespective of macrophage polarisation status (Fig. 5a). Next, NK-mediated ADCC was assessed using hPBMC as effector cells and Ramos cells as targets. The rituximab hIgG1 μtp pre-formed hexamer and hIgG1 μtp C575S retained efficient ADCC activity when compared with their hIgG1 wild-type counterpart (Fig. 5b). These results were mirrored with BHH2 constructs (Supplementary Figure 8b and c). These results demonstrate that FcγR-mediated effector function is not impacted after fusion of the μtp at the C-terminus.

Anti-CD20 mAb also possesses the ability to elicit DCD with differing mechanisms of action, being either more apoptotic (rituximab) or lysosomal (BHH2)^2,37^. To investigate the impact of the hIgG1 μtp pre-formed hexamer and hIgG1 μtp C575S on DCD for these two antibody types, Raji cells were incubated with mAb at various concentrations for 24 h and cell death assessed by flow cytometry (Supplementary Figure 9a). Rituximab hIgG1 wild-type displayed an inherently low ability to induce DCD, which was not enhanced with the addition of the μtp C575S, however, the hIgG1 μtp pre-formed hexamer caused a significant increase in DCD (Fig. 5c). Conversely, the BHH2 hIgG1 wild-type had an efficient induction of DCD in its non-modified form, which was decreased with the μtp C575S and further decreased with the μtp pre-formed hexamer (Fig. 5d). The results, therefore, indicate that μtp modifications can modulate DCD according to the nature of the associated mAb; for type I CD20 mAb increasing it but for type II CD20 mAb reducing it, presumably through receptor re-orientation/hexamerisation in both cases.

Next, we assessed the impact of our μtp fusion in whole-blood B-cell depletion assays. These assays provide a more complete set of physiological effectors, as well as being able to evaluate an overall impact from multiple contributors^38^. B-cell depletion in whole blood was calculated using the ratio of CD3+ T cells to CD19+ B cells after incubation with anti-CD20 mAb (Supplementary Figure 9b). Surprisingly, given its powerful CDC activity, the rituximab hIgG1 μtp pre-formed hexamer displayed a significant decrease in B-cell depleting efficacy compared to the hIgG1 μtp C575S (58% decrease) and hIgG1 wild-type (62% decrease) formats (Fig. 5e), which were equivalent. A similar trend was observed with BHH2 reagents (Fig. 5f), which demonstrated higher B-cell cytotoxicity than rituximab reagents overall, and a 31 and 37% decrease for BHH2 hIgG1 μtp pre-formed hexamer compared with BHH2 hIgG1 μtp C575S and BHH2 hIgG1 wild-type, respectively. These data indicate that hIgG1 μtp C575S-mediated on-target hexamerisation does not improve or hinder effector functionality but that pre-formed hexamers reduce killing activity in the context of multiple potential effector mechanisms, with a larger impact with more complement-active mAb such as rituximab. To address whether the μtp formats exhibited differential activity for the high (158 V) or low (158 F) FcγRIIIa polymorphisms^39,40^, we genotyped the same samples. We observed no unexpected effects, with the μtp pre-formed hexamer being least effective in FcγRIIIa V/V, V/F and F/F donors and μtp C575S, exhibiting the same efficacy as wild-type IgG1 across the genotypes (Supplementary Figure 9c).

### Analysis of in vivo B-cell depletion

**Fig. 6:**
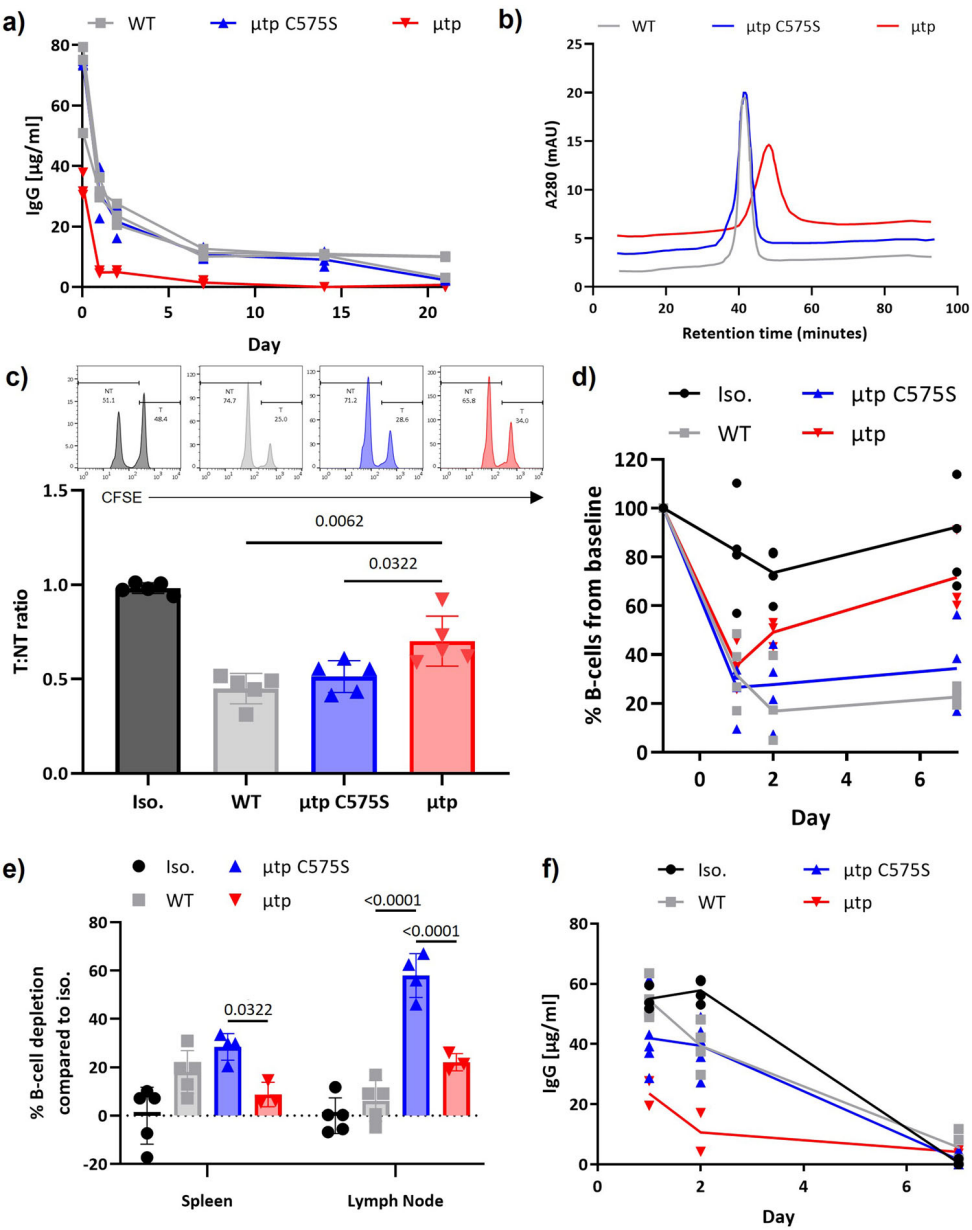
In vivo B-cell depletion using the rituximab IgG1 μtp fusion mAb. **a** Balb/C mice were administered 100 μg rituximab hIgG1 μtp constructs i.v. and peripheral serum was collected at 2 h and days 1, 2, 6, 14, and 21. The concentration of mAb in the serum was calculated by ELISA (*n* = 3). **b** FcRn binding was analysed by loading hIgG1 μtp constructs onto an FcRn affinity column at 1 mg/ml pH 5.5, and eluting using a pH gradient up to pH 8.8. **c** A 1:1 ratio of CFSE-labelled hCD20 Tg splenocytes (high) and wt splenocytes (low) were adoptively transferred into C57 BL/6 mice i.v. followed 24 h later by 25 μg rituximab hIgG1 mAb constructs i.p. After 24 h mice were sacrificed and splenocytes stained for B220. B-cell depletion was calculated using a T:NT ratio of CFSE high (T) to CFSE-low (NT) B cells in the spleen of treated mice (*n* = 5). **d** hCD20 Tg Balb/C mice were administered 100 μg rituximab hIgG1 mAb constructs i.v. on day 0. Circulating B-cell levels were monitored on days 1, 2, and 7 by peripheral blood collection using CD19/B220 flow cytometry staining. B-cell depletion is expressed as a % of B cells compared to pre-mAb administration (*n* = 5). **e** Spleen and inguinal lymph nodes were harvested on day 15, and B-cell depletion was assessed using CD19/B220 flow cytometry staining (*n* = 5). **f** Serum samples collected at each time point were used to determine circulating IgG concentration by ELISA (*n* = 5). Individual data points and mean are plotted from biologically independent animals. Statistical analysis was carried out by one-way ANOVA.

Finally, we assessed the activity of these various μtp constructs in mice. Antibody clearance was investigated in wild-type mice lacking any human CD20, to remove confounding issues relating to target binding. The rituximab hIgG1 μtp C575S mAb displayed a similar rate of antibody persistence compared with the rituximab hIgG1 wild-type, being readily measurable past 2 weeks. Conversely, the μtp pre-formed hexamer was far more rapidly cleared from the serum, with >90% lost within 2 days (Fig. 6a). To probe whether this was related to inadequate binding to FcRn, we measured the binding to FcRn using affinity chromatography. The hIgG1 μtp C575S mAb displayed comparable FcRn retention compared with wild-type hIgG1, whereas the μtp demonstrated stronger retention to FcRn potentially owing to its higher avidity (Fig. 6b).

Next, we assessed B-cell depletion, initially using a previously described adoptive transfer assay^41^. A 1:1 ratio of CFSE high hCD20 Tg B cells and CFSE-low wild-type B cells were transferred into recipient wild-type mice and depletion induced by anti-CD20 μtp mAb monitored in the spleen (Supplementary Figure 10a). The rituximab hIgG1 μtp pre-formed hexamer demonstrated a small but significant decrease in efficacy compared to rituximab hIgG1 wild-type and μtp C575S, which had comparable depletion (Fig. 6c). These results were confirmed with the type II BHH2 hIgG1 μtp pre-formed hexamer. BHH2 demonstrated the highest efficacy in adoptive transfer assays as expected^41^, but further emphasised the reduction in B-cell depletion with the μtp pre-formed hexamer (Supplementary Figure 11). To further evaluate in vivo potency, we assessed systemic B-cell depletion over time in hCD20 Tg Balb/C mice (Supplementary Figure 10b). Rituximab hIgG1 μtp C575S displayed similar B-cell depleting activity to rituximab hIgG1 wild-type and suppressed circulating B cells for 7 days, following a single 100 μg dose (Fig. 6d). In contrast, whereas initially the rituximab hIgG1 μtp pre-formed hexamer displayed a high capacity to deplete peripheral B cells, B-cell numbers recovered more quickly from 24 h post administration (Fig. 6d). On day 15, the animals were killed and organs analysed by flow cytometry to ascertain the extent of B-cell depletion. In the spleen, B-cell depletion was highest with the rituximab hIgG1 μtp C575S mutant, although not significantly different than wild-type hIgG1, whereas the rituximab hIgG1 μtp pre-formed hexamer was significantly less effective (Fig. 6e). In the lymph node, this trend was more pronounced with both wild-type and μtp pre-formed hexamer treated groups displaying significantly (approximately fivefold) less B-cell depletion than the μtp C575S-treated groups (Fig. 6e). The concentration of mAb was also measured in the serum, which demonstrated comparable rates of IgG clearance for the wild-type and µtp mAb. However, the µtp pre-formed hexamer exhibited lower concentrations compared with the monomeric hIgG during the experiment (Fig. 6f).

## Discussion

There is clear evidence that hexamerisation-enhanced hIgG1 formats can augment complement activation above the natural IgG molecule and that enhanced complement activation remains a goal for certain therapeutic mAb^15,16,26^. One example where complement activation is considered beneficial is with the anti-CD20 mAb, ofatumumab. Ofatumumab offers enhanced complement activation above that seen with rituximab, likely owing to its unique and more surface proximal binding epitope and/or low off-rate^42–44^ and has been observed to increase tumor cell elimination in vivo^45^ and achieve clinical responses in patient’s refractory to rituximab monotherapy^46^. In addition, there are benefits to enhancing the complement activity of mAb-targeting bacterial cells, where studies have demonstrated that bacterial infections can be controlled by complement, but not FcγR-mediated effector mechanisms^47,48^.

When designing strategies to enhance CDC, there are two conventional routes; enhancing affinity for C1q^25,49^ or avidity^15^. The latter can be accomplished by pre-formed antibody hexamerisation or on-target antibody hexamerisation. On-target hexamerisation presumably involves non-covalent Fc–Fc interactions initiated after cell surface antigen binding^15^. High avidity Fc interactions are now considered critical for efficient recruitment of C1q^21,24,50^, and by extension induction of CDC. This has been exploited with the use of a single E430G mutation^16^, which has demonstrated broad applicability against a range of cellular targets^15,47,51^. A recent study has further demonstrated that superior CDC can also be induced through the formation of hetero-hexamers (mixed specificity hexameric antibody complexes) that act synergistically, and that these activities are further enhanced by hexamerisation-enhanced mutations, such as E430G^52^. Here, we explored alternative means to elicit on-target hexamerisation using μtp formats.

The resulting hIgG1 μtp pre-formed hexamer molecules exhibited a large (130-fold) enhancement in CDC activity above wild-type hIgG1, with the μtp C575S format providing more modest enhancements. The μtp C575S CDC enhancement required cell surface binding and was not an inherent property of the molecule, unlike the pre-formed hIgG1 μtp hexamer, which exhibited increased C1q binding in ELISA. These activities correlated with the propensity to hexamerise in solution, presumably through non-covalent hydrophobic interactions between µtp’s, similar to those proposed in multimeric IgM structures^20,53^. In solution, self-association was restricted to high concentration for μtp C575S formats (e.g., >20 mg/ml), and was fully reversible with no aggregates, similar to results reported for E430G mutations^16^. The μtp C575S monomer exhibited wild-type binding to C1q and failed to evoke spontaneous activation of serum complement, in contrast to the μtp pre-formed hexamer, which liberated C4d. When bound to target antigen at the cell surface the μtp C575S demonstrated increased C1q recruitment, indicating enhanced avidity, in-line with the concept of on-target hexamerisation.

The same enhancement in complement activation was also observed with hIgG1 μtp molecules containing P331S, hIgG4 μtp and to a lesser extent hIgG2 μtp, demonstrating that hIgG molecules that have low-to-no native affinity for C1q^31,32^ can recruit C1q and activate complement when in a favourable multimeric conformation, i.e., a pre-formed hexamer. This observation is broadly in-line with CDC activity of hIgG2 and hIgG4 µtp hexamers shown by Smith et al.^14^, although here we fully purified the µtp pre-formed hexamer from the monomeric fraction. The presence of fully purified hIgG µtp pre-formed hexamers highlights the benefit gained from higher avidity interactions with C1q provided by a hexameric format, in the absence of high C1q affinity. However, this augmentation was less obvious when the C575S mutation was introduced into hIgG2 and hIgG4 constructs, indicating that this format cannot overcome the inherent isotype disadvantages with regard to C1q binding^32^. The lack of detected C1q recruitment with hIgG2/4 monomeric constructs, but observed Ramos cell killing highlights the highly sensitive nature of this cell line to complement. Unexpectedly, the addition of the μtp C575S to hIgG4 resulted in a loss in complement efficiency, the cause for which has not been fully elucidated.

Accordingly, the effectiveness of the hIgG1 μtp C575S in augmenting CDC was partly dictated by the nature of the target and specific mAb employed as exemplified by the differences in the magnitude of CDC enhancement between type I and type II anti-CD20 mAb. Type I anti-CD20 reagents trigger reorganisation of CD20 into lipid raft microdomains^33^, facilitating mAb clustering and higher levels of CDC owing to a more favourable Fc distribution^30^. Conversely, type II anti-CD20 mAb does not elicit CD20 redistribution, leading to lower CDC activity^30^. The pre-formed hIgG1 μtp hexamer was highly effective in engaging C1q and evoking CDC, indicating that by adopting the hexameric format, previously CDC-inert reagents can be engineered to exhibit potent complement activity (similar to the results with sub-optimal hIgG isotypes detailed above). Interestingly, the μtp hIgG1 C575S fusion was to some extent able to overcome type II characteristics. In this hIgG1 context, the C1q affinity is measurable and so subsequent “on-target” hexamerisation is able to elicit increased C1q binding and CDC.

We also showed that the hIgG1 μtp C575S could elicit a CDC enhancement against a second clinically relevant haematological cell target. The anti-CD38 antibody daratumumab, approved for use against multiple myeloma, was augmented after fusion to either the μtp C575S or μtp, demonstrating that this technology can be applied to a wider range of targets beyond CD20. In addition to haematological cancer cell targets, the killing of HER-2+ SK-BR-3 solid tumor cells with the trastuzumab hIgG1 μtp hexamer indicated that this technology could provoke increased CDC towards targets outside CD20 and malignancies outside of lymphoma. The lack of enhanced CDC activity for the trastuzumab hIgG1 μtp C575S over the hIgG1 likely reflects the greater complement resistance of these targets and is possibly a result of the high expression of complement regulatory proteins CD46, CD55 and CD59^54^.

This differential effect of the μtp fusions on alternative mAb frameworks was also seen with type I versus II anti-CD20 mAb with regards DCD, where hIgG1 hexamers evoked increased DCD with rituximab but reduced DCD with BHH2. The redistribution of CD20 into lipid rafts by type I mAb enables its engagement with a host of BCR signalling proteins, followed by apoptosis^55^. The enhanced DCD observed with the rituximab μtp construct is consistent with studies showing receptor clustering and apoptosis are enhanced through hyper-cross-linking IgG^56^. In contrast, type II anti-CD20 mAb elicit high levels of DCD without the requirement for CD20 clustering or increased IgG cross-linking, by evoking a non-apoptotic, non-autophagic cell death involving actin cytoskeleton remodelling^2,37^. This activity was reduced upon IgG hexamerisation, indicating that the optimal bivalent binding geometry of type II anti-CD20 mAb is disrupted when hexameric. Such differences likely relate directly to their alternate binding geometries^57^ and recently defined differing F(ab):receptor complexes^58^.

Importantly, the addition of the μtp or μtp C575S did not have any impact on ADCC and ADCP. Although the binding avidity of hIgG1 μtp hexamers for FcγR at the cell surface appeared to be enhanced, especially for FcγRIIa and FcγRIIb, the lack of improvement in FcγR-effector functionality suggests that these multimeric Fc formats do not augment FcγR engagement or activation, which is not unexpected given the 1:1 stoichiometry of Fc:FcγR binding^59–62^ and supported by the lack of enhanced binding affinity when measured by SPR.

Although the pre-formed hexameric μtp reagents exhibited significantly enhanced CDC activities in vitro, they displayed reduced efficacy in whole-blood deletion assays ex vivo and in B-cell depletion assays in vivo. The loss of activity in vivo is likely explained by the rapid clearance of the μtp hIgG1 from the serum. Although not directly studied, this rapid clearance presumably relates to its size (~870 KDa) and/or hexameric conformation, which would be expected to be removed either through filtration^63,64^, or via enhanced binding with FcγR^65^. In addition to this potential impairment of deletion, the high avidity interaction with C1q may outcompete FcγR binding, therefore favouring CDC and excluding ADCC, which may be less efficient in killing in the whole-blood assay. This is further evidenced by the larger decrease in B-cell depletion with the rituximab hIgG1 μtp pre-formed hexamers compared with the BHH2 μtp hIgG1 hexamers (58% and 31%, respectively, compared with their μtp C575S counterparts). Alternatively, it can be postulated that an over-activation of CDC may be detrimental to the efficacy of B-cell depletion in blood by impairing other effector mechanisms. This has been previously shown for NK-mediated ADCC through a C3b-dependent downregulation of NK cell binding to IgG immune complexes, resulting in decreased cytotoxicity^66^. Although not previously demonstrated, this same mechanism may impair other FcγR-mediated effector functions such as ADCP.

In contrast to the hIgG1 μtp pre-formed hexamers, the hIgG1 μtp C575S on-target hexamer exhibited modestly increased CDC, coupled to wild-type hIgG1 clearance in the serum and B-cell deletion activity in whole-blood assays. Depletion of peripheral blood B cells in vivo was equivalent between hIgG1 and hIgG1 C575S μtp formats in terms of magnitude and duration. Intriguingly, there was a trend towards increased deletion in the spleen and a highly significant (approximately five-fold) augmentation of B-cell depletion in the lymph node with rituximab hIgG1 μtp C575S. It has previously been shown that complement components are produced in the lymph nodes^67^ and that local complement activation can occur at this site^68^. These data suggest that B-cell depletion in the lymph node may have a higher dependence on complement as opposed to FcγR-mediated mechanisms. In support of this, macrophages and NK cells in lymph nodes express lower levels of activatory FcγRs compared to the spleen and blood^69^, and the overall proportion of macrophages in the lymph nodes is far lower than in the spleen and so it may be that CDC has a greater role in deleting target cells at this site.

Clearly, there are several important aspects to consider when engineering IgG for increased complement activation. The greatest bioavailability of complement is in the circulation, therefore it could be perceived that complement-engineered mAb will have the highest impact in haematological malignancies and in environments that are well-vascularised. However, a recent report has indicated that such hexamerisation-enhanced mAb even have efficient CDC activity under conditions of limiting complement availability^70^. In addition to the potential question of complement bioavailability, there are limitations in the use of mouse models to study complement-enhanced mAb. For example, in our previous studies, we demonstrated that complement plays a limited role in rituximab-mediated B-cell depletion in vivo for canonical IgG mAb^71^.

In conclusion, here, we report an antibody engineering strategy focussed on antibody hexamerisation delivered through the addition of a small 18 amino-acid peptide of human antibody origin. Choice of such a “natural” sequence may limit immunogenicity. The hIgG1 μtp pre-formed hexamers and hIgG1 μtp C575S “on-target” hexamers show enhanced complement effects, through increased C1q avidity. Whereas the hIgG1 μtp pre-formed hexamer is more active in the absence of target binding, the hIgG1 μtp C575S on-target hexamer mAb was only more active after target binding. These mAb exhibit enhanced CDC activity in vitro whilst maintaining other mechanisms of target deletion, such as ADCC and ADCP. Ablating covalent hexamerisation with the C575S mutation reduced the potency of CDC activation but also obviated the negative impacts associated with covalent IgG hexamers; notably purification challenges, spontaneous complement off-target activation, increased IgG serum clearance, and FcγR-related safety risks when administered systemically, as previously highlighted with hIgG1 Fc hexamers^29^. The results described here indicate that such a technology could be applied as a generic CDC-enhancing tool for existing direct targeting mAb with a range of cell surface targets, such as CD20 or EGFR to augment their efficacy and improve anti-cancer therapy.

## Methods

### Cell lines and animals

Ramos and Raji cells were obtained from ATCC. CHO cells stably transfected with human FcγR were produced in-house. All cells were cultured in complete Roswell Park Memorial Institute (RPMI) (Thermo Fisher Scientific) supplemented with 2 mM l-glutamine, 1 mM pyruvate, and 10% fetal calf serum, unless otherwise stated. CHO-SXE cells (UCB proprietary cell line)^72^ were maintained in CD-CHO media (Thermo Fisher Scientific) supplemented with 6 mM l-glutamine. Mice were bred and maintained in local facilities and experiments approved by the local ethical committee under Home Office license PPL30/2964, reporting to the Home Office Animal Welfare Ethical Review Board (AWERB) at the University of Southampton. Experiments conformed to the Animal Scientific Procedure Act (UK).

### Antibody production and quality control

Human IgGs were each directly fused at their C-terminal lysine residues to the 18 amino-acid wild-type (PTLYNVSLVMSDTAGTCY) or mutant (PTLYNVSLVMSDTAGTSY) human IgM μtp. DNA constructs were ordered from ATUM and C575S mutagenesis carried out using Quikchange Lightning Site-directed mutagenesis (Agilent). Constructs were transfected into CHO-SXE cells using ExpiFectamine CHO transfection kit (Thermo Fisher Scientific) according to the manufacturer’s High Titre protocol. Transfected CHO cells were cultured for 10 days. The supernatant was harvested by centrifugation at 4000 ⨯ *g* for 40 mins and clarified by filtration through a 0.22 μm stericup filter. hIgG monomers and hexamers were purified by MabSelectSure Protein A affinity chromatography column (GE Healthcare), followed by size-exclusion chromatography (SEC) using a HiLOad Superdex 200 16/60 GL column (GE Healthcare). Purified antibodies were analysed for purity, endotoxin, MW, and epitope binding.

### Analysis of antibody yield by protein G chromatography

In order to estimate the protein yield for each mAb construct, 100 μl of expression supernatant was loaded onto a 1 ml HiTrap Protein G column (GE Healthcare) attached to an HPLC Infinity System (Agilent). Bound antibody was washed with 20 mM NaPO_4_, 50 mM NaCl pH 7.4, and eluted with 50 mM glycine pH 2.7. The eluted protein absorbance at 280 nm was measured and the area under the peak calculated. Protein yield was calculated using a standard curve calculated from the elution profile of an IgG standard.

### Purity analysis

To determine the purity of each mAb construct, SEC was used. For SE-HPLC 20 μg of protein was loaded onto a TSKgel G3000SWxl gel filtration column (Tosoh Bioscience) attached to the HPLC Infinity System. Protein was eluted over 17 mins with a 0.2 M phosphate buffer pH 7.0 at a flow rate of 1 ml/min. The absorbance at 280 nm was analysed and protein purity was calculated by peak integration and measurement of the area under each peak. For SE-UPLC, 1 µg purified protein sample was injected onto an ACQUITY BEH200 column (Waters) and developed with an isocratic gradient of 200 mM phosphate, pH 7.0 at 0.35 ml/min. Signal detection was by absorbance at 280 nm and multi-channel fluorescence. For quality control purposes all hIgG µtp C575S monomeric mAb was required to have a purity >98% and hIgG µtp pre-formed hexameric mAb a purity >95%.

### Endotoxin analysis

Endotoxin was measured using the Endosafe^®^ Portable Test System (Charles River) or the Limulus Amebocyte Lysate chromogenic endotoxin quantification kit (Pierce), according to the manufacturer’s instructions to ensure endotoxin levels of all antibodies were <1.5 EU/mg.

### SEC-MALS

To determine the absolute molecular weight of the expressed constructs SEC-MALS was used. In all, 50 μg of protein was loaded onto a pre-equilibrated Superdex 200 increase 10/300 column (GE Healthcare), and eluted isocratically using phosphate-buffered saline (PBS) pH 7.4 at 0.5 ml/minute over 60 mins. The column was attached to an Agilent 1100 HPLC system, connected in series to a Viscotek MALS 20 multi-angle light scattering detector and refractive index (RI) detector. The RI detector was calibrated using bovine serum albumin (BSA) and the molecular weight of the proteins of interest calculated using OmniSEC software (Malvern).

### SDS-PAGE

SDS-PAGE analysis was used to assess protein purity of hIgG monomers and hexamers. NuPAGE 3–8% tris-acetate gels used to analyse hIgG μtp hexamers. In all, 2 μg of protein was prepared with Novex Tris-Acetate SDS running buffer and either 10 nm *N*-ethylmaleimide (Thermo Fisher Scientific) or 10% NuPAGE sample reducing agent (Thermo Fisher Scientific) and denatured at 95°C for 10 mins. NativeMark molecular weight marker (Thermo Fisher Scientific) for Tris-Acetate was used. Gels were stained with InstantBlue protein stain according to the manufacturer’s instructions (Expedeon).

### Negative stain electron microscopy

Antibody (10 μg/ml) was applied to electron microscopy grids with 2% uranyl acetate solution and allowed to dry. Electron microscope images were acquired using a Hitachi HT7700 Transmission Electron Microscope at ×80,000 magnification. Images were processed using Adobe Photoshop.

### ELISA

In all, 96-well MaxiSorp plates (NUNC) were coated with the appropriate protein at the required concentration, serially diluted across and coated at 4°C overnight. Unbound protein was removed and plates were blocked with PBS 1% BSA before the addition of protein or serum, and incubated at 37°C for 90 mins, followed by detection using horseradish peroxidase (HRP)-conjugated detection antibody. After washing, o-phenylnediamine dihydrochloride was added and the reaction ended with H_2_SO_4_ after an appropriate colour change. Absorbance was measured at 450 nm on an Epoch plate reader (Biotek).

To assess the binding affinity of C1q to mAb, plates were coated with the appropriate mAb at serial dilutions from 10 μg/ml. Following coating and blocking, 2 μg/ml of human purified C1q was added and incubated for 2 h at room temperature. In the case of the C1q ELISA, a primary rabbit anti-C1q antibody (Abcam) was added next and incubated, followed by the HRP-conjugated donkey anti-rabbit IgG (Sigma) detection antibody.

In order to determine the concentration of hIgG1 in the peripheral blood of mice after administration, plates were coated with goat-anti-human antibody (gamma chain specific) (Sigma-Aldrich) at serial dilutions from 100 μg/ml. Following coating and blocking, serum samples were added to the plate at an initial dilution of 1:100 or matched controls at a starting concentration of 1 μg/ml and serially diluted across the plate, and incubated at 37°C for 90 mins. Following incubation, the HRP-conjugated F(ab’)_2_ goat-anti-human (Fc specific) (Jackson Immunoresearch) detection antibody was added.

### Fluid-phase C4 activation

Complement C4 activation in human serum was determined by measuring the concentration of C4d. hIgG1 constructs (100 μg/ml) were incubated in normal human serum (NHS) for 1 h at 37°C. C4d concentration was then measured by ELISA (MicroVue EIA C4d, Quidel) according to the manufacturer’s protocol.

### C1q recruitment analysis

To determine C1q recruitment to the cell surface, 1 × 10^5^ CD20+ Ramos target were opsonised with hIgG µtp constructs at concentrations between 10 and 0.15 µg/ml for 15 mins at room temperature. Purified human C1q (Abcam) was then added to a final concentration of 2 μg/ml and incubated at 37°C for 10 mins. The cell mixture was washed with fluorescent activated cell sorting (FACS) wash before staining for bound C1q with anti-C1q-FITC (Abcam) and incubated for 30 mins at 4°C before analysis by flow cytometry (BD FACS Calibre).

### CDC assay

NHS was prepared from the blood of healthy volunteers with appropriate consent. Venous blood was taken into glass vials to clot. Clotted blood was centrifuged at 900 × *g* for 20 mins and collected serum stored in glass vials at −80 °C. For the CDC assay, CD20+ Ramos or Raji cells were opsonised with mAb at the desired concentrations for 30 mins at 4°C. NHS was then added at 20% V/V and incubated for 30 mins at 37°C. Cell death was measured as propidium iodide (PI) positive cells (%) by flow cytometry (BD FACS Calibre).

### SPR analysis

SPR was carried out to assess the binding affinities of mAb to FcγR using a Biacore T100 system (GE Healthcare). A Series S Sensor CM5 chip (GE healthcare) was primed and normalised with BIA Normalising solution (GE Healthcare). The normalised chip dextran was activated with a 1:1 mixture of EDC (0.4 M 1-ethyl-3-(3-dimethylaminopropyl)-carbodiimide) and NHS (0.1 M N-hydroxysuccinimide) (Amine Coupling kit; GE Healthcare) for 10 mins. The mAb ligand was diluted to 25 μg/ml in Acetate pH 5 (GE Healthcare) and ~2000 response units (RU) were immobilised to the CM5 sensor chip flow cells via amine chemistry. Ethanolamine (Amine Coupling kit; GE Healthcare) was used to deactivate excess dextran groups on the chip flow cells. Recombinantly produced FcγR (I, IIA, IIB, IIIA, IIIB) (R&D Systems) were prepared in HBS-EP (GE Healthcare) at 0.16–100 nM (FcγRI) or 1.6–1000 nM (FcγRIIa, IIb, IIIa, IIIb). Kinetic analysis was performed according to the following parameters: sample on/off times 300 s at a flow rate of 30 μl/min with 30 s regeneration of 30 μl/min 10 mM Glycine pH 2.0. FcγR flowed through all cells simultaneously. A blank reference cell was used to be subtracted from antibody-containing flow cells. Kinetic analysis and steady-state affinity calculation were performed using Biacore Evaluation software (GE Healthcare).

### Flow cytometry

For direct detection of cell surface proteins, cells were incubated with fluorescently labelled antibodies for 30 mins at 4°C or 15 mins at room temperature. Labelled cells were washed twice with FACS wash (PBS, 1% w/v BSA (Europa Bioproducts), 0.01% v/v sodium azide (Sigma-Aldrich) and centrifuged at 300 × *g* for 5 mins. For indirect detection of hIgG bound to the cell surface, target cells were opsonised with mAb for 30 mins at 4°C or 15 mins at room temperature, washed twice with FACS wash, 0.01% sodium azide (Sigma-Aldrich) at 300 × *g* for 5 mins, and labelled with mouse anti-human IgG-APC (clone M1310G05) for 30 mins at 4°C or 15 mins at room temperature. Labelled cells were washed twice with FACS wash at 300 × *g* for 5 min. Samples were analysed using a FACS Calibre or Canto (Becton Dickinson) and data analysis was performed using FlowJo (Becton Dickson).

### FcγR binding

mAb were incubated with 1 × 10^5^ CHO cells stably expressing different human FcγR^36^ for 30 mins at 4°C, followed by washing with FACs wash and detection with PE-anti-human IgG F(ab’)_2_ (Jackson ImmunoResearch). The binding of mAb was assessed by flow cytometry.

### FcRn binding

mAb were buffer exchanged into 20 mM MES-HCl pH 5.5, 140 mM NaCl and adjusted to 1 mg/ml before loading onto an FcRn affinity column (Roche Custom Biotech) equilibrated with 80% 20 mM MES-HCl pH 5.5, 140 mM NaCl and 20% 20 mM Tris-HCl pH 8.8, 140 mM NaCl. Bound antibody was eluted over 30 column volumes using a pH gradient by increasing the percentage of 20 mM Tris-HCl pH 8.8, 140 mM NaCl, and measured by absorbance at 280 nm.

### PBMC isolation

Human peripheral blood mononuclear cells (PBMC) were isolated from blood leucocyte cones (acquired from Southampton General Hospital NHS Blood Service) diluted in PBS, ethylenediaminetetraacetic acid (EDTA; 2 mM). Diluted blood was layered onto Lymphoprep (Axis Shield) and centrifuged at 800 × *g* for 10 mins. The interphase layer containing the PBMCs was collected and washed with PBS, EDTA three times before resuspension in appropriate media at an appropriate concentration.

### ADCC assay

Ramos cells at 1 × 10^7^ cells/ml in PBS were labelled with 10 µM Calcein AM (Life Technologies) for 30 mins at 37°C and washed. Cells were then opsonised with antibody for 30 mins at 4°C. PBMCs in complete RPMI were co-cultured with labelled target cells at a ratio of 50:1 effectors to targets for 4 h at 37°C. Lysis buffer (Triton X-100) was used to assess maximum lysis, and untreated Ramos cells incubated with PBMCs as background. Cell death was measured as Calcein release using a Varioskan (ThermoScientific) at 455 nm. The percentage of cell cytotoxicity was measured as follows: ((test RFU—background RFU)/Max lysis RFU—background RFU)) × 100 (RFU = relative fluorescent unit).

### ADCP assay

PBMCs in RPMI supplemented with 1% human AB serum were differentiated into MDMs by adding PBMCs to six-well plates for <2 h at 37°C to allow monocytes to adhere and non-adherent cells removed by washing. Human M-CSF (Peprotech) was added at 50 ng/ml on alternate days and the resulting macrophages used 7 days later. CLL target cells were labelled with 5 μM CFSE for 10 mins at room temperature before washing and resuspending to the appropriate concentration. MDMs were plated at 1 × 10^6^ cells/ml and co-cultured with antibody opsonised CFSE-labelled target cells for 1 h at 37°C at a 5:1 effector to target ratio. MDMs were labelled with FcγRIII-APC (3G8) and scraped off the plate to be transferred into FACs tubes. Phagocytosis was assessed by measuring the proportion of FcγRIII + MDMs that stained positive for CFSE via flow cytometry using FACs Calibur. To polarise macrophages, 200 ng PAM3CSK4 (M1) or 80 ng IL4 and 16 ng IL13 (M2) was added at 24 h intervals for 48 h prior to use.

### DCD assay

Target cells at 1 × 10^6^ cells/ml were incubated with antibody at 37 °C for 24 h in cRPMI. Direct cell death was measured by the percentage of double-positive PI and Annexin-V-FITC (produced in-house) cells by flow cytometry (FACS Calibur).

### Whole-blood B-cell depletion assay

Blood from healthy human volunteers was drawn into lithium heparin vacutainers (BD Biosciences) and used within 3 h of collection. In all, 237.5 μl of blood was added to 12.5 μl mAb to give a final concentration of 1 μg/ml, and incubated at 37°C for 24 h. Blood was stained with anti-CD45, anti-CD19, and anti-CD3 before lysing with FACsLysis Solution (BD Biosciences) and analysed by flow cytometry (FACS Canto). B-cell depletion was calculated by analysing the ratio of B cells to T-cells to derive a cytotoxicity index (CTI): 100− ([100/(%CD19+/%CD3+)] × [%CD19 control/%CD3 control]).

### In vivo adoptive transfer B-cell depletion assay^73^

In vivo B-cell depletion was assessed using an adoptive transfer model where whole splenocyte suspensions from hCD20 Tg C57 BL/6 and wild-type C57 BL/6 mice labelled with 5 mM and 0.5 mM CFSE, respectively, and mixed in a 1:1 ratio. In all, 5 × 10^6^ cells injected intravenously into recipient wild-type C57 BL/6 mice. Mice received 25 μg mAb 24 h later intraperitoneally, and spleens were harvested 18 h later. Spleen suspensions were labelled with APC-anti-CD45R and analysed by flow cytometry (FACS Calibur) to determine the ratio of target (T) to non-target (NT) cells.

### In vivo B-cell depletion

Female human CD20 transgenic (Tg) Balb/C mice (aged 3–6 months) were treated with 100 μg mAb i.v. on day 0. The number of B cells remaining in blood or organs was then assessed by flow cytometry (FACS Calibur) for CD19-PE and CD45R-ACP positive B cells. Residual B-cell numbers in treated mice were calculated as a percentage of baseline B cells, recorded 1 day prior to mAb administration.

### Biological materials

Unique biological materials will be made available upon reasonable request or can be produced de novo by researchers using the amino-acid sequences that can be made available upon request using standard mammalian cell production and antibody purification techniques.

### Statistics and reproducibility

Data were processed using GraphPad Prism and one-way and two-way analysis of variance statistical test used to analyse two or more independent, continuous data groups.

### Reporting summary

Further information on research design is available in the Nature Research Reporting Summary linked to this article.

## Supplementary information

Supplementary Information

Reporting Summary

## Data Availability

The data sets generated during and/or analysed during the current study are available from the corresponding author on reasonable request. Unprocessed blots are included in supplementary figures (Supplementary Figure 12).
