# Supplementary material for: On-target IgG hexamerisation driven by a C-terminal IgM tail-piece fusion variant confers augmented complement activation
Source: Commun Biol. 2021 Sep 2;4:1031. doi: 10.1038/s42003-021-02513-3 (PMC8413284; doi:10.1038/s42003-021-02513-3)
Supplement: Supplementary file 1 — Supplementary Information [file 42003_2021_2513_MOESM1_ESM.pdf]

Format	V-region	Expressed yield (mg/L)	Purified yield (mg/L)	% of expressed yield
IgG1 WT	RTX	364.2	323.6	88.9
	BHH2	326.9	68.0	20.8
	Trastuzumab	123	128.9	104.8
	mAb X	112.4	103.1	91.7
	mAb Y	333.0	61.0	18.3
IgG1 µtp C575S	RTX	391.1	104.0	26.6
	BHH2	395.0	267.6	67.7
	Trastuzumab	278.0	97.8	35.2
	mAb X	286.0	180.4	63.1
	mAb Y	382.9	286.7	74.9
IgG1 µtp	RTX	359.6	64.0	17.8
	BHH2	131.1	84.4	64.4
	Trastuzumab	216.0	17.8	8.2
	mAb X	80.0	37.8	47.3
	mAb Y	250.0	45.0	18

Supplementary Table 1: Estimated expression yield and calculated purified yield for IgG1 µtp constructs containing different V regions. Expression yield (mg/L) was calculated post expression by protein G HPLC and purified yield (mg/L) was calculated post size exclusion chromatography. Constructs containing 5 different 5 regions were assessed.

Format	V-region	Expressed yield (mg/L)	Purified yield (mg/L)
IgG2 WT	RTX	302	28
IgG2 µtp C575S	RTX	69	14
IgG2 µtp	RTX	168	6
IgG4 WT	RTX	741	99
IgG4 µtp C575S	RTX	437	72
IgG4 µtp	RTX	530	26

Supplementary Table 2: Estimated expression yield and calculated purified yield for RTX IgG2 and IgG4 µtp constructs. Expression yield (mg) was calculated post expression by protein A HPLC and purified yield (mg) was calculated post size exclusion chromatography.

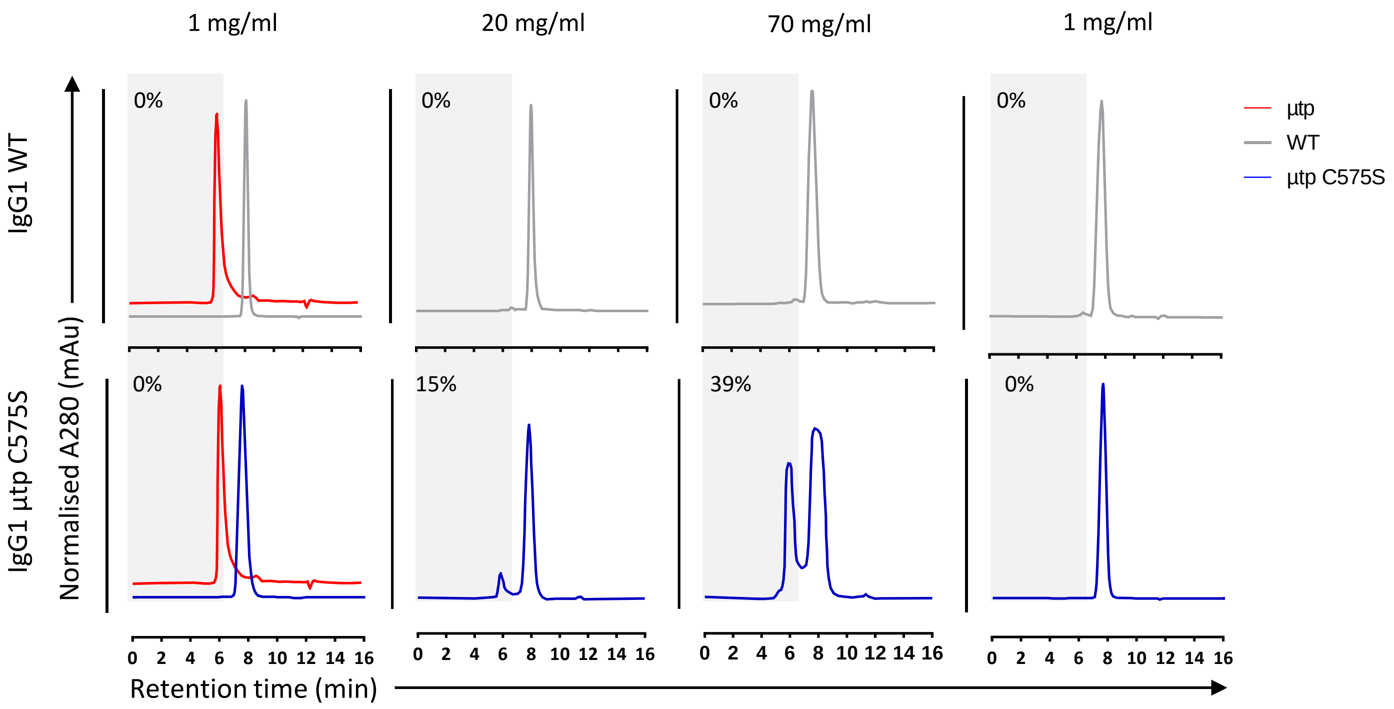

Supplementary Figure 1: Trastuzumab hIgG1 constructs were concentrated up to 70 mg/ml and diluted to the required concentrations and analysed by SE-HPLC for the percentage of monomeric and multimeric species. The left column row shows hIgG1 WT (top) and hIgG1 µtp C575S (bottom) overlayed with purified hIgG1 µtp pre-formed hexamer trace prior to concentration. The middle two traces represent concentration to 20 mg/ml and 70 mg/ml, respectively and the right trace is post dilution back to 1 mg/ml of hIgG1 WT (top) and hIgG1 µtp C575S (bottom).

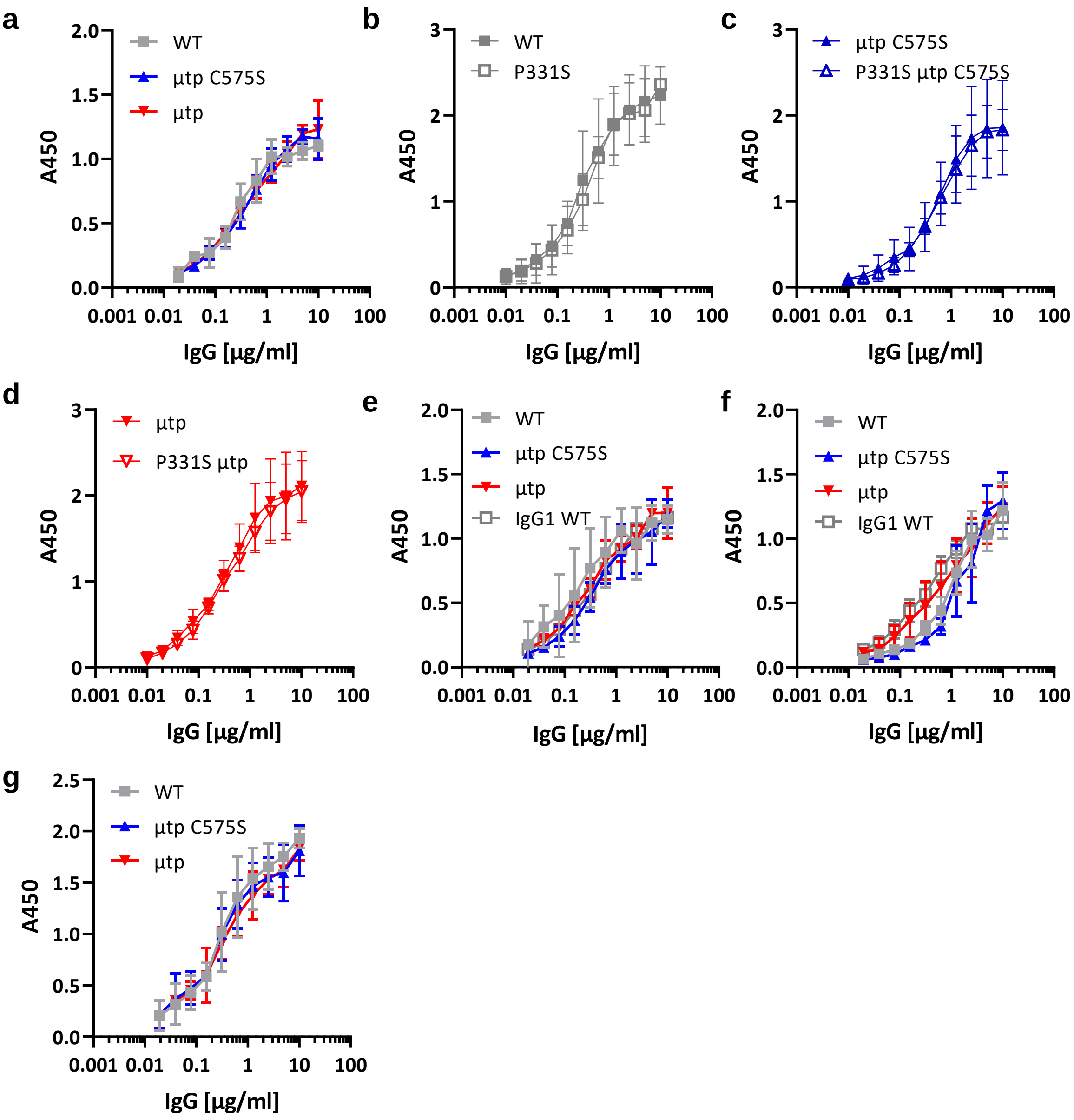

Supplementary Figure 2: Elisa plates were coated with the desired antibody at various concentrations and detected with an HRP-conjugated goat anti-human IgG to confirm similar levels of antibody was bound to the plate compared to the C1q binding plate. Plates have been coated with **a)** RTX hIgG1 **b)** RTX hIgG1 P331S **c)** RTX hIgG1 P331S µtp C575S **d)** RTX hIgG1 P331S µtp **e)** RTX hIgG2 **f)** RTX hIgG4 **g)** BHH2 hIgG1 antibodies. Data depicts mean and SD from independent experiments (n=3).

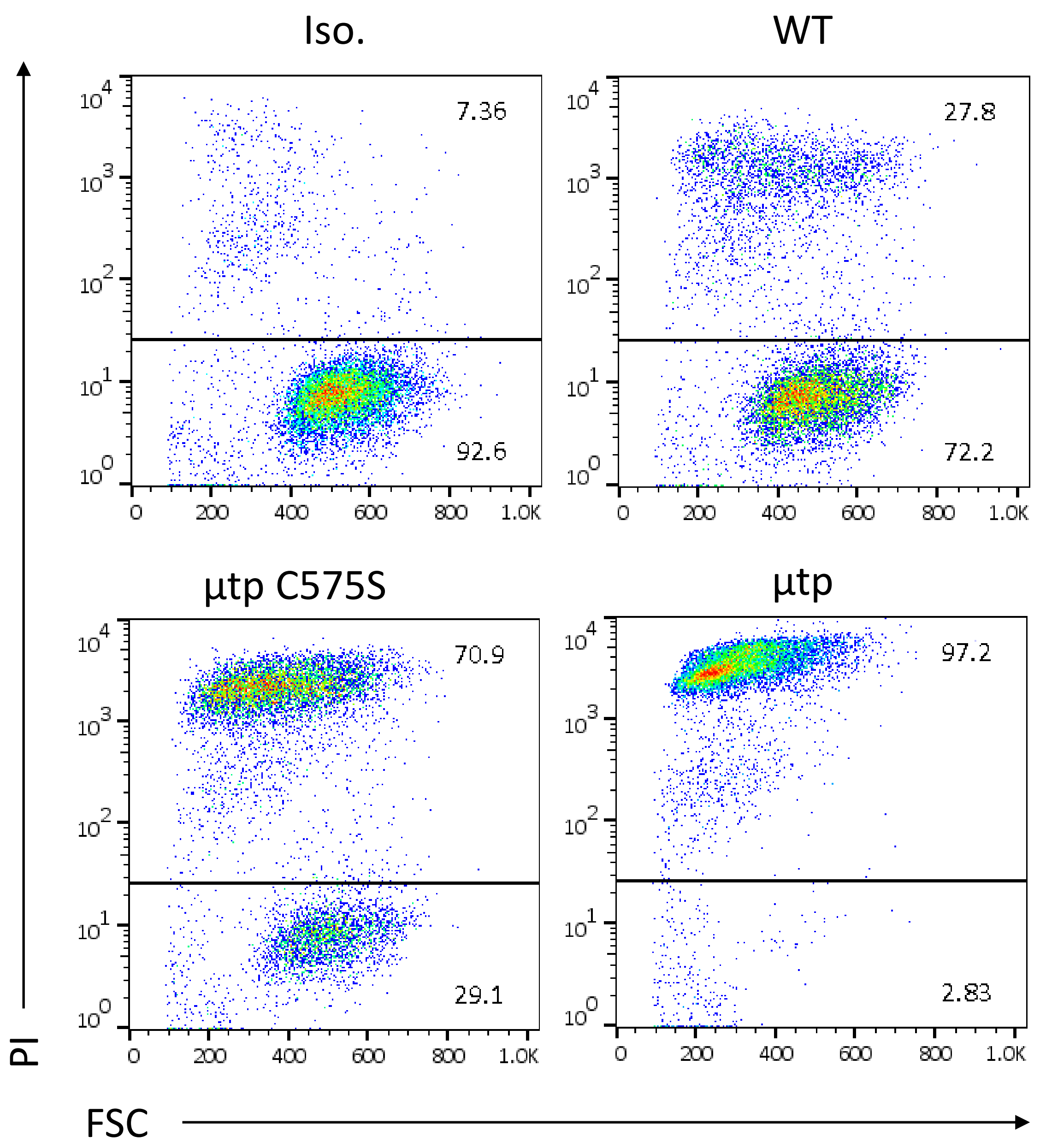

Supplementary Figure 3: Flow cytometry evaluation of in vitro CDC activity. Raji cells were opsonised with RTX hIgG1 µtp constructs at a range of concentrations and incubated with NHS (20 % V/V). Cell death was examined as the percentage of PI positive cells by flow cytometry. Data shows cell death at 10 µg/ml.

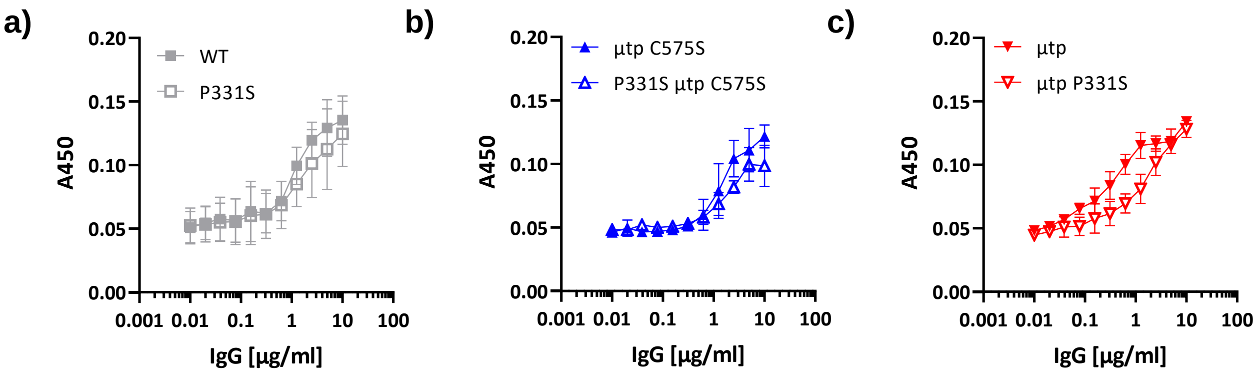

Supplementary Figure 4: C1q binding to µtp P331S. C1q binding to µtp P331S. C1q binding for µtp P331S constructs was measured using ELISA. ELISA plates were coated with hIgG µtp constructs at various concentrations and purified human C1q (2 µg/ml) added. Bound C1q was detected with a goat-anti-C1q, followed by an anti-goat-HRP conjugated antibody. Data shows absorbance at 450 nm. Data showing mean and SD for WT **a)**, µtp C575S **b)** and µtp **c)** (n=3).

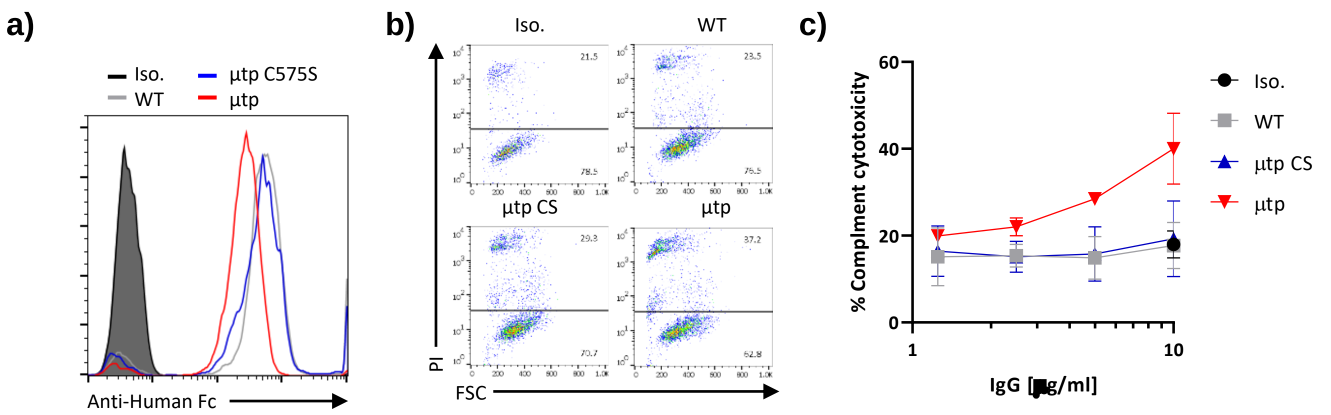

Supplementary Figure 5:CDC enhancement is also observed with a trastuzumab IgG1 µtp construct. **a)** SK-BR-3 cells were opsonised with trastuzumab hIgG µtp mAb at 10 µg/ml and binding measured by secondary anti-human Fc-FITC labelled antibody. **b)** CDC-induced cell death of trastuzumab hIgG1 µtp constructs was assessed by opsonising SKBR-3 cells and incubating with NHS (20 % v/v). Cell death was examined as the percentage of PI positive cells by flow cytometry. **c)** Cell death of SK-BR-3 cells was assessed by CDC over a concentration range. All data shown is mean and SD from independent experiments (n=3).

a

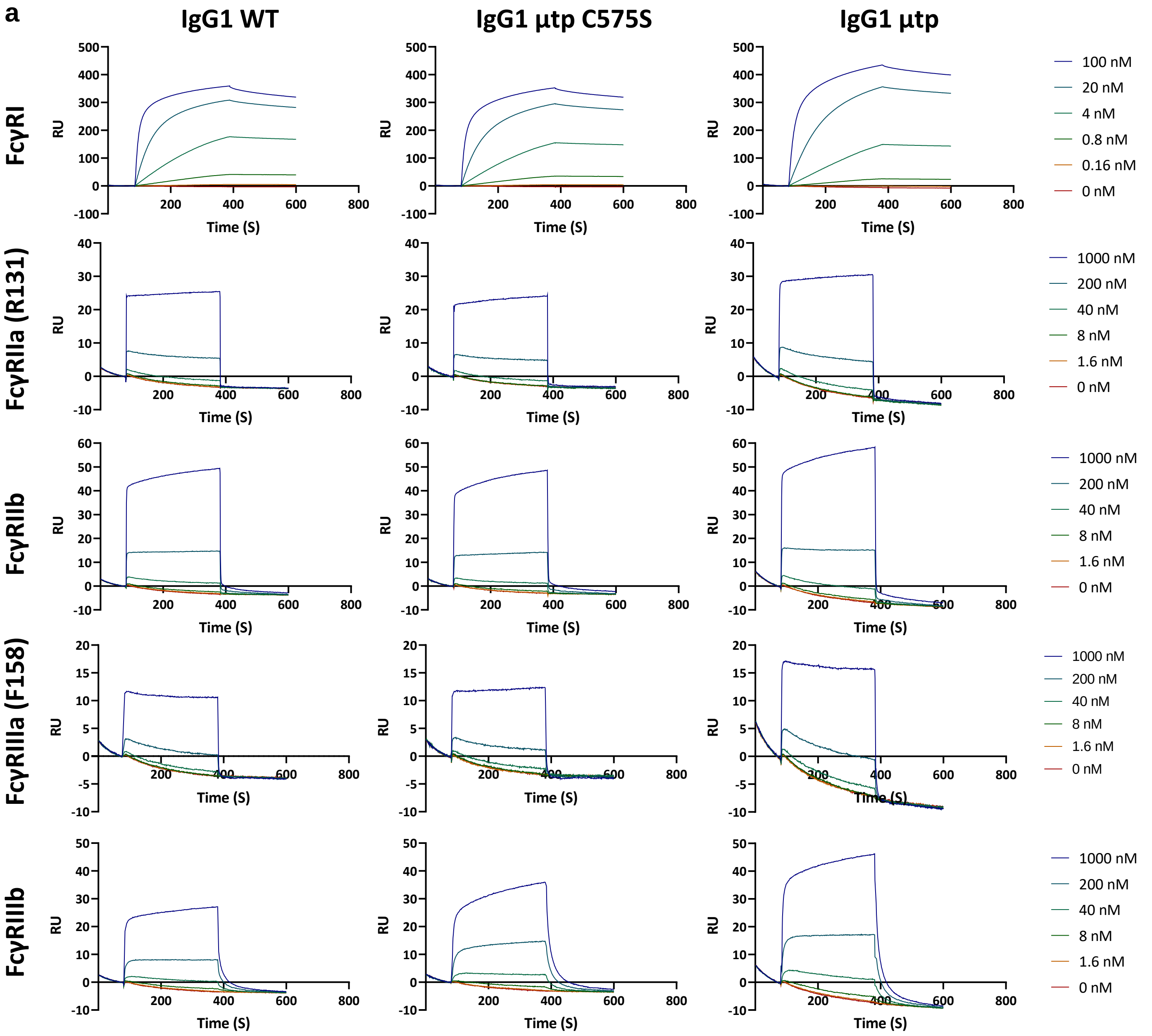

b

	KD (nM)		
Receptor	IgG1 WT	IgG1 µtp C575S	IgG1 µtp
FcγRI	4.36	5.33	7.53
FcγRIIa (R131)	935	1000	964
FcγRIIb	1360	1540	1470
FcγRIIIa (F158)	719	415	410
FcγRIIIb	1860	1680	1510

Supplementary Figure 6: Affinity analysis of RTX hIgG1 µtp mAb binding human FcγR. **a)** Antibodies were immobilised to a Biacore binding chip and recombinant FcγR's flowed over for 300 seconds followed by 300 seconds of dissociation at several concentrations. Binding of FcγR to immobilised mAb was measured in response units. Representative data shown of a single experiment. **b)** Affinities were calculated using a steady state affinity model. Representative data shown of a single experiment.

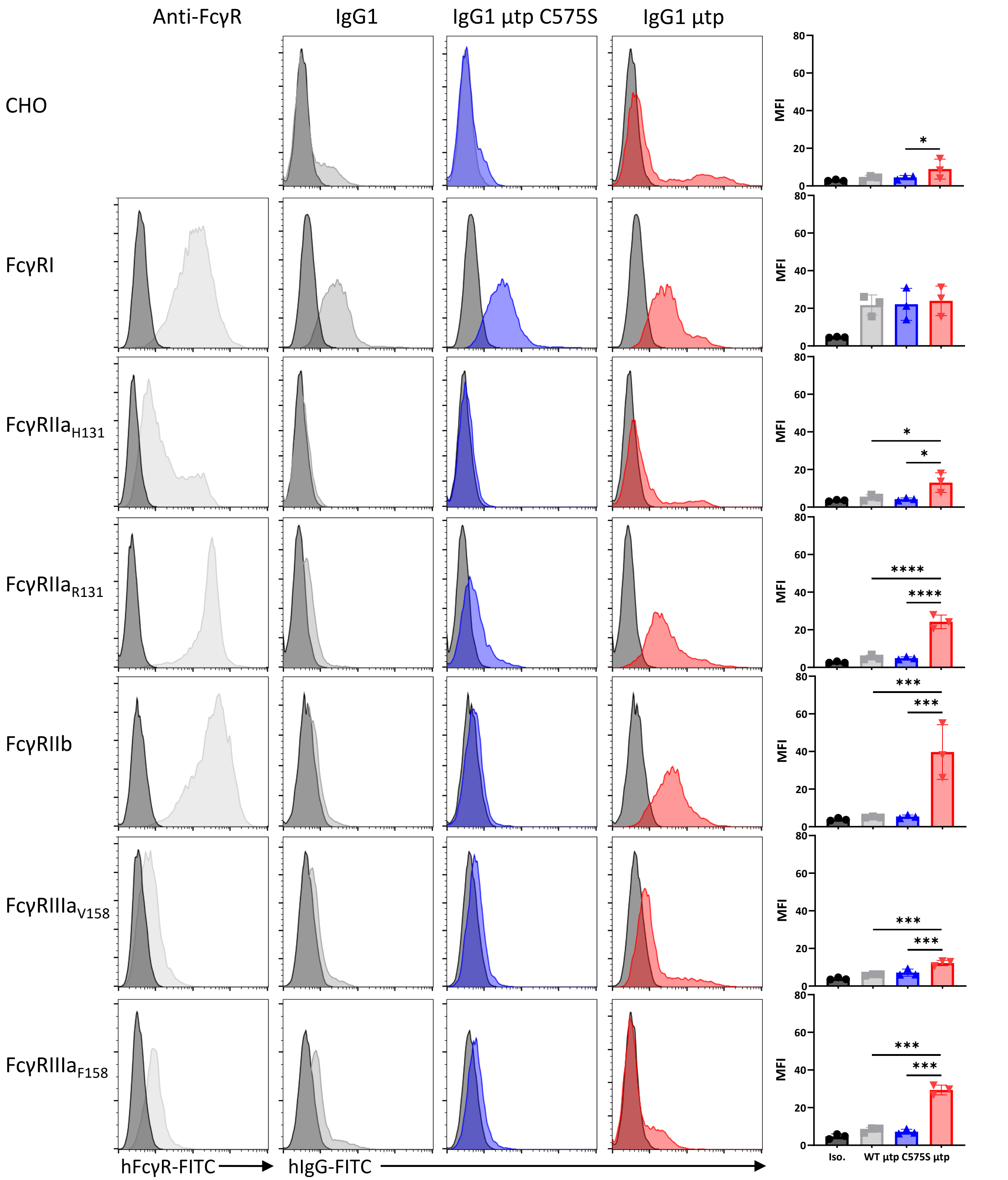

Supplementary Figure 7: Analysis of the FcγR binding capabilities of hIgG1 µtp mAb. CHO cells stably expressing different human FcγR were used to assess the level of RTX hIgG1 µtp mAb binding at the cell surface. Representative FcγR expression pattern on the surface of transfected cells (left column) using cell surface staining (Isotype - solid black; FcγR expression – light grey) with 10.1-FITC (FcγRI), AT10-FITC (FcγRII) 3G8-FITC (FcγRIII). Transfected CHO cells were also incubated with RTX hIgG1 µtp (middle columns). Bound mAb was detected by anti-human $F(Ab')_2$ (PE) and analysed by flow cytometry. The graphs on the right show the mean and SD of MFI from independent experiments (n=3). Statistics calculated using one-way ANOVA. * $P \leq 0.05$, *** $P \leq 0.001$, **** $P \leq 0.0001$.

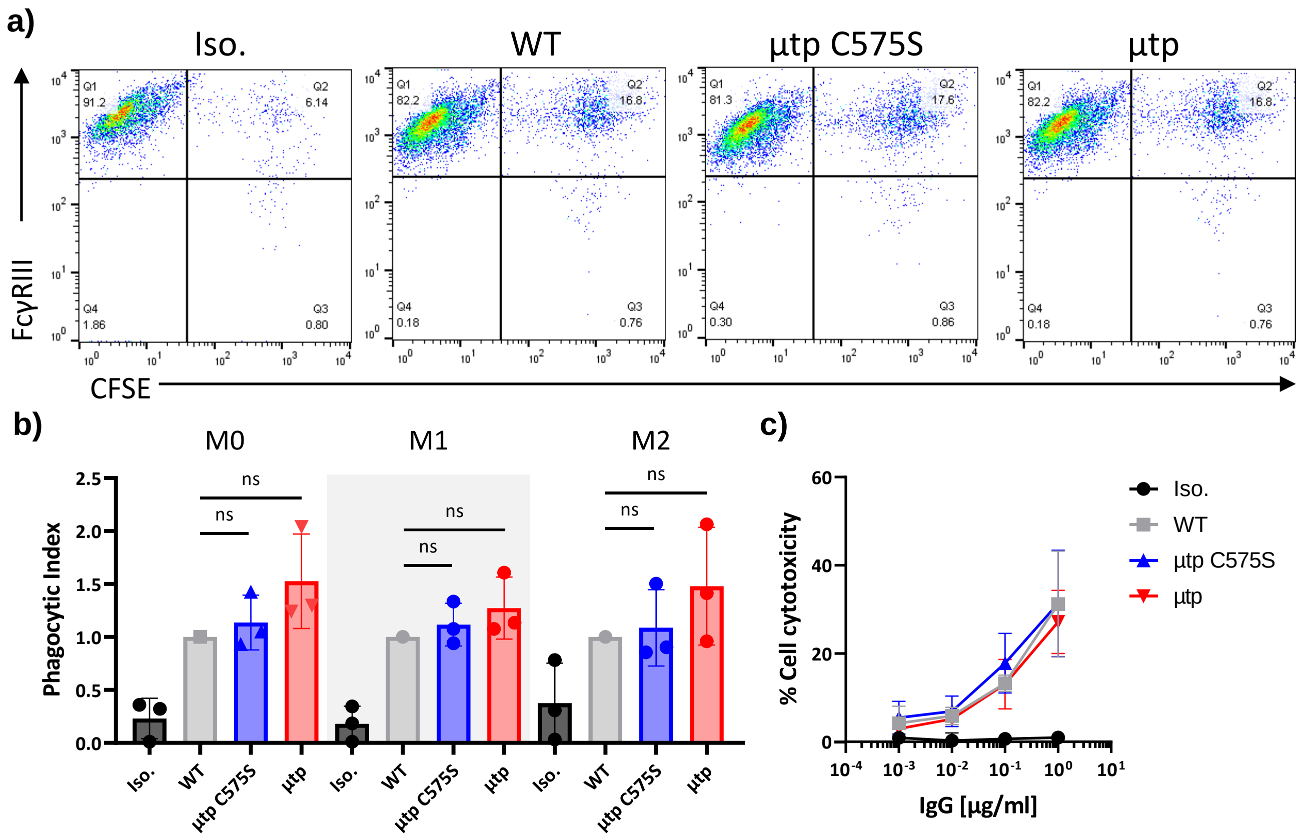

Supplementary Fig. 8: Assays to assess FcγR-mediated effector functions in vitro. a) CLL PBMCs, labelled with CFSE, were opsonised with 0.5 µg/ml RTX IgG1 µtp constructs and co-cultured with human MDMs. Phagocytosis was measured by flow cytometry assessing double positive macrophages for CFSE and FcγRIII by flow cytometry. **b)** CLL PBMCs, labelled with CFSE, were opsonised with 0.5 µg/ml BHH2 hIgG1 µtp constructs and co-cultured with human MDMs. Phagocytosis was measured by flow cytometry assessing double positive macrophages for CFSE and FcγRIIIa by flow cytometry. Phagocytosis was examined in macrophages skewed in vitro to M0, M1 (Pam3SK4 stimulation), and M2 (IL4/IL13 stimulation) polarisation states. Data shows the phagocytic index mean and SD from independent experiments (n=3). **c)** Calcein labelled Ramos cells were opsonised with BHH2 hIgG1 µtp constructs and incubated with freshly purified PBMCs. The calcein release from cells was used to calculate the % of cell cytotoxicity (n=3). Data plotted as mean and SD of independent experiments. Statistical analysis was carried out by one-way ANOVA.

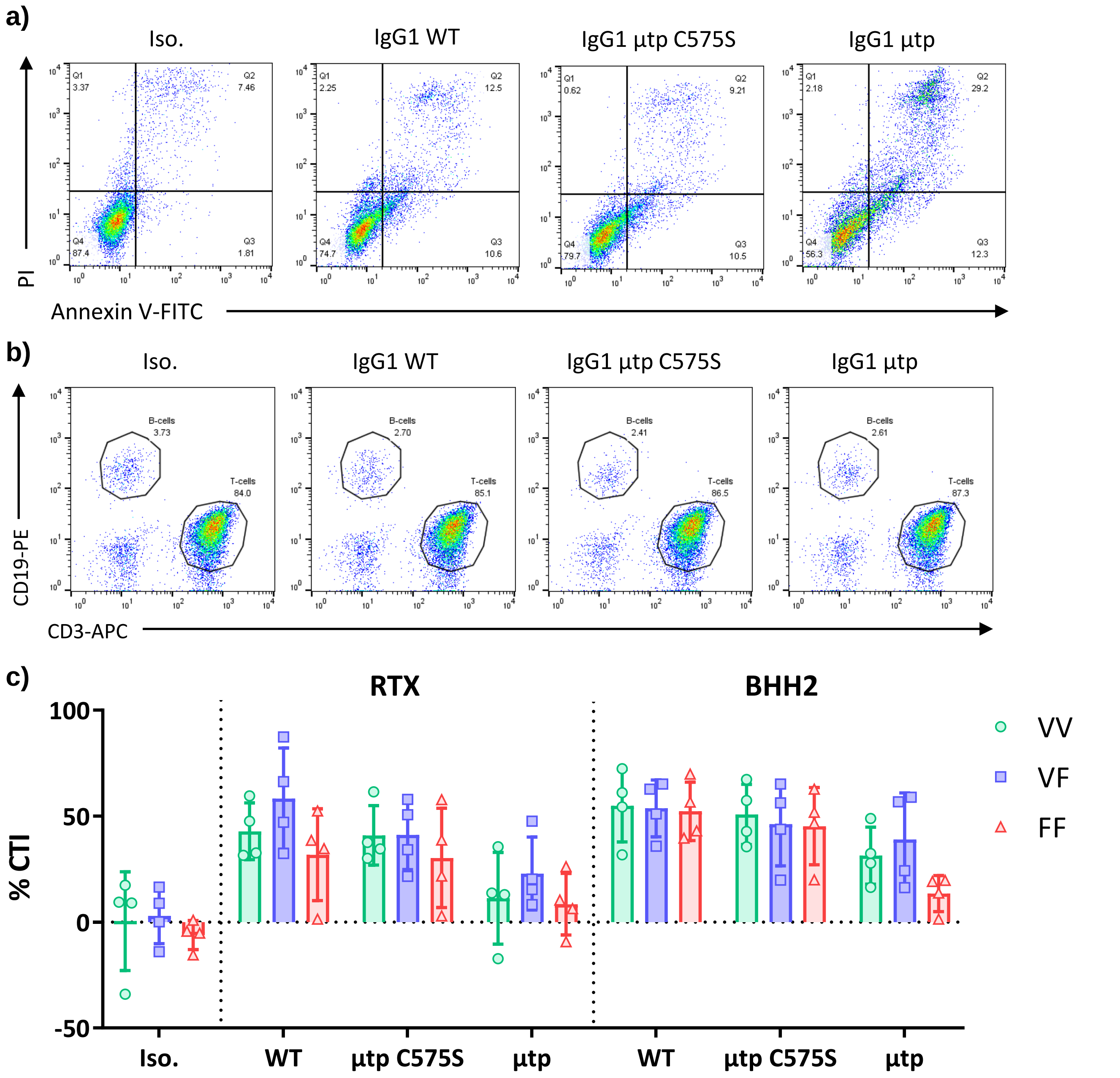

Supplementary Figure 9: Cell death and whole blood B cell depletion of RTX IgG1 µtp fusion mAb. a) For direct cell death, Raji target cells were incubated with RTX hIgG1 µtp for 24 hours at 37ºC. DCD was assessed by annexin V/PI flow cytometry. **b)** For the whole blood B cell depletion assay, fresh peripheral human blood was incubated with RTX IgG1 µtp fusion mAb (1 µg/ml) for 24 hours at 37ºC. B cell depletion (Cytotoxicity index (CTI)) was calculated by the ratio of B cells to T cells by flow cytometry. Flow cytometry analysis of CD3+ T cells and CD19+ B cells in blood treated with RTX hIgG1 µtp fusion mAb. **c)** Fresh peripheral human blood from genotyped donors was incubated with IgG1 µtp fusion mAb (1 µg/ml) for 24 hours at 37ºC. B cell depletion (Cytotoxicity index [CTI]) was calculated by the ratio of B cells to T cells using flow cytometry.

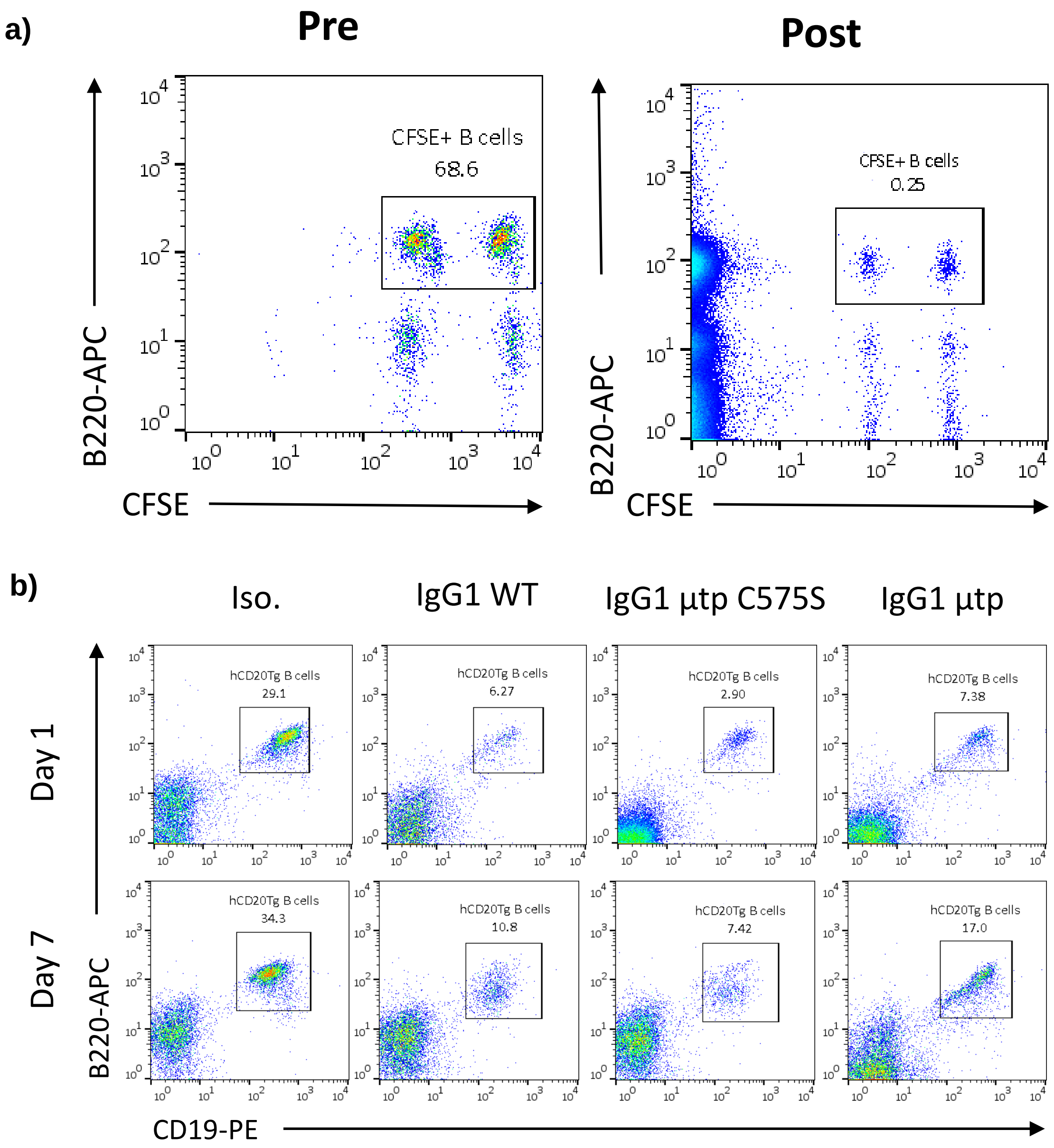

Supplementary Figure 10: FACS analysis for in vivo depletion with RTX hIgG1 contructs. a) A 1:1 ratio of CFSE labelled hCD20Tg splenocytes (high) and wt splenocytes (low) were adoptively transferred into C57 BL/6 mice i.v. followed 24 hours later by 25 µg RTX hIgG1 mAb constructs i.p. After 24 hours mice were sacrificed and splenocytes stained with B220 to analyse the depletion of hCD20Tg B cells. **b)** hCD20Tg Balb/C mice were administered 100 µg RTX hIgG1 mAb constructs i.v. on day 0. Circulating B cell levels were monitored on days 1, 2, and 7 by peripheral blood collection using CD19/B220 flow cytometry staining.

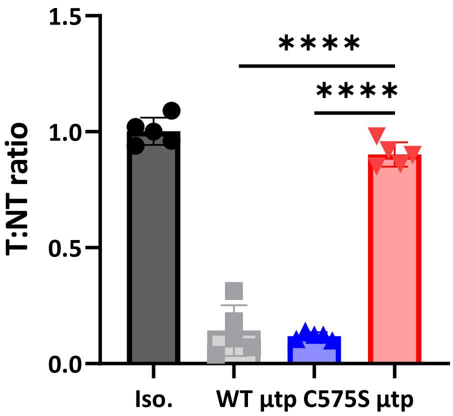

Supplementary Figure 11: Analysis of in vivo B cell depletion of BHH2 hIgG1 µtp fusion mAb of adoptively transferred hCD20Tg splenocytes. A 1:1 ratio of hCD20Tg splenocytes labelled with 5 µM CFSE (high) and wt splenocytes labelled with 0.5 µM CFSE (low) were adoptively transferred into C57 BL/6 mice i.v. followed 24 hours later by 25 µg BHH2 hIgG1 µtp constructs i.p.; 24 hours later mice were sacrificed and splenocytes were stained with B220 to analyse the depletion of hCD20Tg CFSE high B cells. B cell depletion was calculated by monitoring the CFSE high (T) to CFSE low (NT) B cells cells in the spleen of treated mice, expressed as the T:NT ratio (n=5). Data are plotted as mean and SD. Statistical analysis was carried out by one-way ANOVA; **** = P<0.0001.
