# Supplementary material for: On-target IgG hexamerisation driven by a C-terminal IgM tail-piece fusion variant confers augmented complement activation
Source: Commun Biol. 2021 Sep 2;4:1031. doi: 10.1038/s42003-021-02513-3 (PMC8413284; doi:10.1038/s42003-021-02513-3)
Supplement: Supplementary file 2 — Reporting Summary [file 42003_2021_2513_MOESM2_ESM.pdf]

Corresponding author(s): Mark Cragg

Last updated by author(s): Jul 16, 2021

Reporting Summary

Nature Research wishes to improve the reproducibility of the work that we publish. This form provides structure for consistency and transparency in reporting. For further information on Nature Research policies, see our Editorial Policies and the Editorial Policy Checklist.

Statistics

For all statistical analyses, confirm that the following items are present in the figure legend, table legend, main text, or Methods section.

n/a	Confirmed	
☐	☒	The exact sample size (*n*) for each experimental group/condition, given as a discrete number and unit of measurement
☐	☒	A statement on whether measurements were taken from distinct samples or whether the same sample was measured repeatedly
☐	☒	The statistical test(s) used AND whether they are one- or two-sided<br>*Only common tests should be described solely by name; describe more complex techniques in the Methods section.*
☒	☐	A description of all covariates tested
☐	☒	A description of any assumptions or corrections, such as tests of normality and adjustment for multiple comparisons
☐	☒	A full description of the statistical parameters including central tendency (e.g. means) or other basic estimates (e.g. regression coefficient) AND variation (e.g. standard deviation) or associated estimates of uncertainty (e.g. confidence intervals)
☐	☒	For null hypothesis testing, the test statistic (e.g. *F*, *t*, *r*) with confidence intervals, effect sizes, degrees of freedom and *P* value noted<br>*Give P values as exact values whenever suitable.*
☒	☐	For Bayesian analysis, information on the choice of priors and Markov chain Monte Carlo settings
☒	☐	For hierarchical and complex designs, identification of the appropriate level for tests and full reporting of outcomes
☒	☐	Estimates of effect sizes (e.g. Cohen's *d*, Pearson's *r*), indicating how they were calculated

Our web collection on statistics for biologists contains articles on many of the points above.

Software and code

Policy information about availability of computer code

Data collection	BD CellQuest and BD FACSDIVA was used to collect flow cytometry data<br>SkanIt software was used to collect flourescent readings for the ADCC assay<br>Agilent Chemstation, Empower3 Chromatography software was used to collect HPLC traces<br>ImageQuant was used to capture SDS-PAGE images<br>BioTek Gen5 software was used to collect ELISA absorbances
Data analysis	GraphPad Prism was used for data analysis and to perform statistical tests<br>FlowJo was used to analyse flow cytometry data<br>Agilent Chemstation, Empower3 Chromatography software was used to analyse HPLC traces

For manuscripts utilizing custom algorithms or software that are central to the research but not yet described in published literature, software must be made available to editors and reviewers. We strongly encourage code deposition in a community repository (e.g. GitHub). See the Nature Research guidelines for submitting code & software for further information.

Data

Policy information about availability of data

All manuscripts must include a data availability statement. This statement should provide the following information, where applicable:

- Accession codes, unique identifiers, or web links for publicly available datasets
- A list of figures that have associated raw data
- A description of any restrictions on data availability

The datasets generated during and/or analyzed during the current study are available from the corresponding author on reasonable request.

Field-specific reporting

Please select the one below that is the best fit for your research. If you are not sure, read the appropriate sections before making your selection.

☒ Life sciences ☐ Behavioural & social sciences ☐ Ecological, evolutionary & environmental sciences

For a reference copy of the document with all sections, see nature.com/documents/nr-reporting-summary-flat.pdf

Life sciences study design

All studies must disclose on these points even when the disclosure is negative.

Sample size	For all in vitro experiments at least three independent repeats were performed to provides sufficient evidence for reproducibility. For in vivo experiments N = 5 was chosen based on previous knowledge of strain and experiment, 5 animals is sufficient for representative results. For the whole blood assay 12 donors were selected due to availability of genotyped donors.
Data exclusions	No data was excluded from the analysis
Replication	In vitro experiments were repeated at least three independent times or with at least three independent donors. In vivo experiments were performed at least 2 independent times.
Randomization	Animals were randomly divided into experimental groups
Blinding	N/A

Reporting for specific materials, systems and methods

We require information from authors about some types of materials, experimental systems and methods used in many studies. Here, indicate whether each material, system or method listed is relevant to your study. If you are not sure if a list item applies to your research, read the appropriate section before selecting a response.

Materials & experimental systems

n/a	Involved in the study	
☐	☒	Antibodies
☐	☒	Eukaryotic cell lines
☒	☐	Palaeontology and archaeology
☐	☒	Animals and other organisms
☒	☐	Human research participants
☒	☐	Clinical data
☒	☐	Dual use research of concern

Methods

n/a	Involved in the study	
☒	☐	ChIP-seq
☐	☒	Flow cytometry
☒	☐	MRI-based neuroimaging

Antibodies

Antibodies used	Rituximab, BHH2, Herceptin, and Daratumumab IgG-IgM tailpiece fusion antibodies were generated in house. The following antibodies were used for flow cytometry: hIgG (M1310G05, Biolegend), mCD19 (1D3, in-house), mCD45r(B220) (RA3-6B2, e-bioscience, 17-0452-82), hC1q (polyclonal, Abcam, ab182940), hCD45 (HI30, BD Biosciences, 555485), hCD3 (UCHT1, BD Biosciences, 561807), hCD19 (HIB19, BD Biosciences, 555413), hCD16 (3G8, in-house), hCD32 (AT10, in-house), hCD64 (10.1, in-house). The following antibodies were used in ELISAs: Donkey anti-Rabbit IgG (Sigma), Goat anti-human IgG (Polyclonal, Jackson immunoresearch, 109-036-008).
Validation	The specificity of commercial antibodies was verified by the manufacturer, in-house antibodies were verified using cell lines specific to their species specificity and data in this manuscript supports their specificity.

Eukaryotic cell lines

Policy information about cell lines

Cell line source(s)	Ramos cells (ATCC), Raji cells (ATCC), CHO cells stably transfected with human FcγR were produced in-house, CHOS-XE cells were produced in-house at UCB.
Authentication	Ramos and Raji cell line identity was confirmed using short tandem repeat analysis (Powerplex 16 System, Promega). CHO cells stably transfected with human FcγR were validated in-house using specific antibodies (Tutt et al JI 2015). CHOS-XE cell line: over expression of XBP1(s) and Ero1a at mRNA and protein level was confirmed by RT-PCR and immunoblotting respectively.

Mycoplasma contamination	Mycoplasma test were conducted using the Mycoplasma: MycoAlert Mycoplasma Detection Kit (Lonza) and returned negative results.
Commonly misidentified lines (See ICLAC register)	None were used in this study

Animals and other organisms

Policy information about studies involving animals; ARRIVE guidelines recommended for reporting animal research

Laboratory animals	Balb/C female, aged 3-6 months. Balb/C hCD20Tg female, aged 3-6 months. C57BL/6 female, aged 3-6 months. C57BL/6 hCD20Tg female, aged 3-6 months.
Wild animals	Study did not involve wild animals
Field-collected samples	Study did not involve samples collected from the field
Ethics oversight	Local ethical committee under Home Office license PPL30/2964 and following approval by local ethical committees, reporting to the Home Office Animal Welfare Ethical Review Board (AWERB) at the University of Southampton.

Note that full information on the approval of the study protocol must also be provided in the manuscript.

Flow Cytometry

Plots

Confirm that:

☒ The axis labels state the marker and fluorochrome used (e.g. CD4-FITC).

☒ The axis scales are clearly visible. Include numbers along axes only for bottom left plot of group (a 'group' is an analysis of identical markers).

☒ All plots are contour plots with outliers or pseudocolor plots.

☒ A numerical value for number of cells or percentage (with statistics) is provided.

Methodology

Sample preparation	Cells from mouse spleens and lymph nodes were harvested by dissociated using a cell strainer. All cells were prepared for flow cytometry by centrifugation for 5 minutes at 300g and resuspended in PBS supplemented with 1%BSA
Instrument	Flow cytometry was performed using FACS Calibur and FACS Canto II instruments
Software	For FACS Calibur data was collected using BD Cell Quest and for FACS Canto II data was collected using BD FACSDiva. Data was analysed using FlowJo
Cell population abundance	10,000 cells were collected for cell lines and 10,000 lymphocytes collected for human and mouse primary cell samples gated on their FSC/SCC properties. For adoptive transfer experiments 2000 CFSE positive B cells were collected after lymphocyte gating.
Gating strategy	FSC/SCC gates were based on prior knowledge of the position of cells in a population based on these parameters due to the cells size and granularity. Positive cell populations were selected based on the observation of distinct populations that were stained for the cell marker being probed. For PI and Annexin-IV staining, positive cells were those above the level of non-positive (control) cells.

☒ Tick this box to confirm that a figure exemplifying the gating strategy is provided in the Supplementary Information.
